# Supplementary material for: Molecular-level insights into the electronic effects in platinum-catalyzed carbon monoxide oxidation
Source: Nat Commun. 2021 Nov 25;12:6888. doi: 10.1038/s41467-021-27238-z (PMC8617298; doi:10.1038/s41467-021-27238-z)
Supplement: Supplementary file 1 — Supplementary Information [file 41467_2021_27238_MOESM1_ESM.pdf]

**Supplementary Information**

# Molecular-level insights into the electronic effects in platinum-catalyzed carbon monoxide oxidation

*Wenyao Chen[1], Junbo Cao[1], Jia Yang[2], Yueqiang Cao[1], Hao Zhang[3,4], Zheng Jiang[4,5], Jing Zhang[1], Gang Qian[1], Xinggui Zhou[1], De Chen,[2]✉ Weikang Yuan,[1] Xuezhi Duan[1]✉*

[1]State Key Laboratory of Chemical Engineering, East China University of Science and Technology, 130 Meilong Road, Shanghai 200237, China.

[2]Department of Chemical Engineering, Norwegian University of Science and Technology, Trondheim 7491, Norway.

[3]Institute of Functional Nano & Soft Materials Laboratory (FUNSOM), Jiangsu Key Laboratory for Carbon-Based Functional Materials & Devices, Soochow University, Suzhou 215123, China.

[4]Shanghai Institute of Applied Physics, Chinese Academy of Sciences, Shanghai 201800, China.

[5]Shanghai Synchrotron Radiation Facility, Zhangjiang Lab, Shanghai Advanced Research Institute, Chinese Academy of Sciences, Shanghai 201210, China.

✉email: chen@nt.ntnu.no; xzduan@ecust.edu.cn.

**Supplementary Note 1**

**The quantity (N) and quality ($r_0$) of active sites.**

For structure-sensitive reactions, the specific activity of each site varies with its identity. For instance, the low-coordinated and high-coordinated sites generally give different intrinsic activities. Based on this, the overall catalytic activity (r) could be expressed by $r=\sum TOF_i \times N_i$, where $TOF_i$ and $N_i$ represent the intrinsic activity and the corresponding number of the specific site i, respectively. To simplify this, $TOF_i$ and $N_i$ could be further replaced with $TOF_0$ and N, where $TOF_0$ and N represent the average catalytic activity and number of these different sites, respectively. Hence, the expression of catalytic activity could be written as $r=\sum TOF_i \times N_i= TOF_0 \times N$.

**Supplementary Note 2**

**Mass transfer calculations for CO oxidation over the Pt/CNT-600.**

**(1) Mears criterion for external diffusion**

If $\frac{r_{obs}\rho_b R_p n}{k_c C_{Ab}} < 0.15$, then external mass transfer effects can be neglected.

Where $r_{obs}$ = observed reaction rate, mol/kg$_{cat}$·s

$\rho_b$ = bulk density of catalyst bed, kg/m$^3$

$R_p$ = catalyst particle radius, m

$n$ = reaction order

$k_c$ = external mass transfer coefficient, m/s

$C_{Ab}$ = bulk gas concentration of CO, mol/m$^3$

As a result, $\frac{r_{obs}\rho_b R_p n}{k_c C_{Ab}}$ = [6.82 × 10$^{-3}$ mol/kg$_{cat}$·s] × [1400 kg/m$^3$] × [4.5 × 10$^{-5}$ m] × [1] / ([0.071 m/s] × [0.446 mol/m$^3$] = 0.014 < 0.15

**(2) Weisz-Prater criterion for internal diffusion**

If $C_{WP} = \frac{r_{obs}\rho_c R_p^{\ 2}}{D_{eff} C_s} < 1$, then internal mass transfer effects can be neglected.

Where $r_{obs}$ = observed reaction rate, mol/kg$_{cat}$·s

$\rho_c$ = density of solid catalyst, kg/m$^3$

$R_p$ = catalyst particle radius, m

$D_{eff}$ = effective diffusivity, m$^2$/s

$C_s$ = gas concentration of CO at the external surface of the catalyst, mol/m$^3$

As a result, $C_{WP} = \frac{r_{obs}\rho_c R_p^{\ 2}}{D_{eff} C_s}$ = [6.82 × 10$^{-3}$ mol/kg$_{cat}$·s] × [2100 kg/m$^3$] × [4.5 × 10$^{-5}$ m]$^2$ / ([8.9 × 10$^{-6}$ m$^2$/s] × [0.446 mol/m$^3$]) = 0.007 < 1.

**Supplementary Note 3**

**The calculation of proposed energy barrier (*ΔE*)**

Because the oxygen containing groups (hydroxyl, carboxyl, carbonyl, and ester) could affect the CO oxidation performance, it is not fair to employ the energy barrier of individual oxygen containing group to evaluate the catalytic activity for a given catalyst. Hence, we propose a new criterion of $\Delta E$ by incorporating the effects of all these oxygen containing groups for this evaluation, which can be defined as:

$$\Delta E=\sum(\Delta E_i \times n_i)$$

where $\Delta E_i$ and $n_i$ represent the energy barrier for a given oxygen containing group and its corresponding content, respectively. Taking the Pt/CNT-0 catalyst for example:

The energy barriers for the CO oxidation over the models of Pt-hydroxyl, Pt-carboxyl, Pt-carbonyl, and Pt-ester were calculated as 2.06, 1.86, 1.71 and 1.91 eV, respectively. On the other hand, the contents of hydroxyl, carboxyl, carbonyl, and ester were determined to be 9.86, 4.20, 2.32 and 4.40 wt%, respectively (Supplementary Table 4). Then, $\Delta E$ could be calculated as:

$$\Delta E=\sum(\Delta E_i \times n_i) = 2.06\times9.86\%+1.86\times4.20\%+1.71\times2.32\%+1.91\times4.40\% = 0.405 \text{ eV}$$

Although this is not a precise method for the energy barrier calculation of a given catalyst, it can be still employed to make a comparison of energy barrier among different catalysts.

**Supplementary Note 4**

**The derivation of TOF based on Pt B.E.**

As shown in Supplementary Fig. 31a, $\ln A_i$ exhibits an almost linear dependence on $E_a$, indicating a significant kinetic compensation effect. According to Cremer-Constable relation, this relation could be interpreted as a thermodynamic balance between the activation entropy and activation enthalpy. Based on the transition state theory, these two kinetics parameters serve as the representations of the adsorption and activation of reaction species, respectively. For this reason, the activation properties of reactants ($E_a$) could be well correlated with Pt electronic properties (Pt B.E.) as shown in Supplementary Fig. 31b. On the other hand, to experimentally elucidate the influences of Pt electronic properties on reactants adsorption properties, steady-state isotopic transient kinetic analysis (SSITKA) was conducted to determine the site coverage of surface species and the intrinsic reaction rate under operando conditions. Typically, the reaction temperature and pressure were raised to 100 °C and 1.85 bar, respectively, which were maintained for another 2 h prior to measuring the reaction rate ($r_{100}'$) and TOF' in Supplementary Fig. 31c and Supplementary Table 7. Specifically, the $^{12}CO$ response curves by switching from Ar/$^{12}CO/O_2$ to Kr/$^{13}CO/O_2$ were recorded in Supplementary Fig. 27, based on which the amount of adsorbed CO ($N_{CO}$) as well as site coverage ($\theta_{CO}$) could be calculated in Supplementary Table 7. Moreover, the $O_2$ site coverages ($\theta_{O2}$) were investigated by switching from Ar/CO/$^{16}O_2$ to Kr/CO/$^{18}O_2$ as shown in Supplementary Fig. 28, which exhibits the almost identical $^{16}O_2$ and Ar response curves possibly due to the limited $O_2$ adsorption on the catalyst surface. However, based on the formation of OOCO from adsorbed CO and $O_2$ to produce $CO_2$ and the direct reaction between the adsorbed O and CO to produce $CO_2$, the amount of adsorbed oxygen species, mainly $O_2$, could be estimated from the amount

of generated $CO_2$ due to the large excess of CO over Pt surface. As a result, the oxygen site coverages ($\theta_{oxygen}$) based on $C^{16}O_2$ response curves by switching from Ar/CO/$^{16}O_2$ to Kr/CO/$^{18}O_2$ (Supplementary Fig. 29) were calculated as shown in Supplementary Table 7. As a result, the adsorption behaviors of reaction species ($\theta_{CO}$ and $\theta_{oxygen}$) were further correlated with Pt B.E. in Supplementary Fig. 31d. Obviously, $\theta_{CO}$ decreases with Pt B.E., agreeing well with the decreased adsorption energy by Pt Bader charge, while $\theta_{oxygen}$ exhibits an opposite trend. Hence, these in situ kinetics information give a quantitative description of CO and oxygen adsorption with Pt B.E., consistent with the positively charged Pt weakening the adsorption of CO to provide more active sites for oxygen adsorption as indicated by DFT calculations.

According to the above DFT calculations, the rate-determining step for CO oxidation for these Pt catalysts appears to be formation of OOCO specie from adsorbed CO and $O_2$. Based on this, the catalytic activity of active site (TOF') could be estimated by the expression of TOF'=$k'\times\theta_{CO}\times\theta_{oxygen}$, in which $k'$ is the reaction rate constant. Further combining the Arrhenius equation, $k'=A_0'\times\exp(-E_a/\mathrm{RT})$, yields *Eq. 1*:

$$\ln A_0' = \ln TOF' - \ln\theta_{CO} - \ln\theta_{\mathrm{oxygen}} + E_a / RT \quad (1)$$

As a result, $\ln A_0'$ (logarithm of frequency factor) could be calculated as shown in Supplementary Table 7, and further correlated with Pt B.E. in Supplementary Fig. 31e. According to the transition-state theory, $\ln A_0'$ is proportional to the activation entropy ($\Delta S^{0*}$), which mainly quantifies the freedom loss of reactants species in terms of binding strength with catalyst. To this point, because Pt B.E. could give a good description of the reactants activation behaviors (Supplementary Fig. 31b) and adsorption behaviors (Supplementary Figs. 31d and 31e), it is attempted to combine the influences of Pt B.E. on the activation and adsorption to

derive a new kinetics model to bridge the microscopic properties of Pt active site and the macroscopic catalytic performance. Herein, combining the above linear relationships gives *Eq. 2*:

$$TOF' = a \times \exp(b \times Pt\ B.E. + c) \quad (2)$$

in which *a, b,* and *c* is determined in Supplementary Table 8 at the given condition. As a result, the predicted TOF' (solid line) based on *Eq. 2* exhibits good consistence with the experimental TOF' (red triangle) in Supplementary Fig. 31f.

**Supplementary Table 1.** The textural properties of CNT-0, CNT-200, CNT-400, CNT-600, CNT-800 and CNT-1000.

| Sample | Specific surface area ($m^2 \cdot g^{-1}$) | Pore volume ($cm^3 \cdot g^{-1}$) |
|---|---|---|
| CNT-0 | 202.9 | 1.65 |
| CNT-200 | 228.3 | 1.55 |
| CNT-400 | 210.1 | 1.57 |
| CNT-600 | 207.3 | 1.62 |
| CNT-800 | 214.6 | 1.52 |
| CNT-1000 | 216.9 | 1.58 |

**Supplementary Table 2.** The structural and electronic properties, as well as catalytic activity of Pt/CNT-0, Pt/CNT-200, Pt/CNT-400, Pt/CNT-600, Pt/CNT-800 and Pt/CNT-1000 for CO oxidation (100 $^{o}$C, atmospheric pressure, $P_{CO}$:$P_{O2}$:$P_{Ar}$=1:20:79).

| Catalyst | Pt B.E. (eV) | $r_{100}$ ($mmol_{CO}·mol_{Pt}^{-1}·s^{-1}$) | $d_{Pt}$ (nm) [a] | $N_{tot,H2}$ ($μmol·g_{cat}^{-1}$)* | $TOF_{Pt}$*$10^{-3}$ ($s^{-1}$) [b] | $N_{tot,CO}$ ($μmol·g_{cat}^{-1}$) | $d_{CO}$ (nm) [c] | $TOF_{CO}$*$10^{-3}$ ($s^{-1}$) [d] |
|---|---|---|---|---|---|---|---|---|
| Pt/CNT-0 | 71.60 | 8.6 | 1.2 | 52.11 | 13.4 | 17.76 | 1.3 | 14.7 |
| Pt/CNT-200 | 71.68 | 16.6 | 1.3 | 61.21 | 28.8 | 19.77 | 1.2 | 25.4 |
| Pt/CNT-400 | 71.85 | 32.8 | 1.2 | 63.91 | 51.1 | 23.17 | 1.1 | 42.7 |
| Pt/CNT-600 | 71.90 | 35.6 | 1.2 | 60.68 | 55.5 | 21.88 | 1.1 | 49.1 |
| Pt/CNT-800 | 71.80 | 26.6 | 1.3 | 58.31 | 46.2 | 17.63 | 1.3 | 45.5 |
| Pt/CNT-1000 | 71.74 | 24.2 | 1.2 | 59.73 | 37.7 | 23.36 | 1.1 | 31.2 |

[a] determined from HAADF-STEM measurement.

[b] based on the Pt particle size from HAADF-STEM measurement.

[c] determined from the $^{12}$CO-$^{13}$CO isotopic switches at 100 $^{o}$C.

[d] based on the reversible adsorption of CO from the $^{12}$CO-$^{13}$CO isotopic switches at 100 $^{o}$C.

*Note: It can be seen that the amount of $H_2$ adsorption is higher than that of Pt atoms for each catalyst, which could be attributed to H spillover to carbon support (J. Catal. 1979, 58, 287–295), H diffusion into the bulk (Surf. Sci. 1985, 160, 37–45), or the ability of under-coordinated metal atoms present at the edges and corners of supported particles to bind more than one H (J. Catal. 1972, 24, 367–384). These results are also consistent with the high dispersion of Pt particles over carbon support.

**Supplementary Table 3.** The percentages of Pt species based on XPS Pt 4f spectra for Pt/CNT-0, Pt/CNT-200, Pt/CNT-400, Pt/CNT-600, Pt/CNT-800 and Pt/CNT-1000.

| Catalyst | $Pt^0$ | $Pt^{2+}$ | $Pt^{4+}$ |
|---|---|---|---|
| Pt/CNT-0 | 55% | 25% | 20% |
| Pt/CNT-200 | 60% | 27% | 13% |
| Pt/CNT-400 | 56% | 22% | 22% |
| Pt/CNT-600 | 60% | 18% | 22% |
| Pt/CNT-800 | 61% | 22% | 17% |
| Pt/CNT-1000 | 59% | 23% | 18% |

**Supplementary Table 4.** Electronic conductivity of Pt/CNT-0, Pt/CNT-200, Pt/CNT-400, Pt/CNT-600, Pt/CNT-800 and Pt/CNT-1000.

| Catalyst | Electronic conductivity (S/cm) |
|---|---|
| Pt/CNT-0 | 5.3 |
| Pt/CNT-200 | 9.7 |
| Pt/CNT-400 | 12.8 |
| Pt/CNT-600 | 13.4 |
| Pt/CNT-800 | 14.2 |
| Pt/CNT-1000 | 15.1 |

*Note: It can be seen that the electric conductivity of carbon support increases with temperature of heat treatment ascribed to the elimination of oxygen-containing groups (Carbon 2013, 59, 2–32; Carbon 2016, 96, 174–183; Carbon 2019, 147, 27–34).

**Supplementary Table 5.** The total contents of OCGs, percentages of different OCGs based on XPS *O 1s* spectra, as well as the contents of different OCGs for Pt/CNT-0, Pt/CNT-200, Pt/CNT-400, Pt/CNT-600, Pt/CNT-800 and Pt/CNT-1000.

| Catalyst | OCGs contents (wt%) | Percentages (%) | | | | Contents (wt%) | | | |
|---|---|---|---|---|---|---|---|---|---|
| | | Carbonyl | Hydroxyl | Ester | Carboxyl | Carbonyl | Hydroxyl | Ester | Carboxyl |
| Pt/CNT-0 | 20.78 | 11.15 | 47.44 | 21.17 | 20.23 | 2.32 | 9.86 | 4.40 | 4.20 |
| Pt/CNT-200 | 12.74 | 12.45 | 46.74 | 24.02 | 16.78 | 1.59 | 5.96 | 3.06 | 2.14 |
| Pt/CNT-400 | 10.07 | 21.06 | 31.60 | 33.01 | 14.33 | 2.12 | 3.18 | 3.32 | 1.44 |
| Pt/CNT-600 | 7.97 | 20.01 | 26.16 | 39.73 | 14.09 | 1.60 | 2.09 | 3.17 | 1.12 |
| Pt/CNT-800 | 6.13 | 20.50 | 30.80 | 31.59 | 17.11 | 1.26 | 1.89 | 1.94 | 1.05 |
| Pt/CNT-1000 | 4.61 | 18.97 | 32.33 | 27.35 | 21.36 | 0.87 | 1.49 | 1.26 | 0.99 |

**Supplementary Table 6.** $E_a$ and ln$A_i$ of Pt/CNT-0, Pt/CNT-200, Pt/CNT-400, Pt/CNT-600, Pt/CNT-800, and Pt/CNT-1000.

| Catalyst | $E_a$ (kJ·mol$^{-1}$) | ln$A_i$ |
|---|---|---|
| Pt/CNT-0 | 33.48 | 6.55 |
| Pt/CNT-200 | 31.18 | 6.27 |
| Pt/CNT-400 | 27.47 | 5.66 |
| Pt/CNT-600 | 25.58 | 5.25 |
| Pt/CNT-800 | 24.92 | 4.92 |
| Pt/CNT-1000 | 26.53 | 5.08 |

**Supplementary Table 7.** SSITKA results of Pt/CNT-0, Pt/CNT-200, Pt/CNT-400, Pt/CNT-600, Pt/CNT-800 and Pt/CNT-1000 for CO oxidation. (100 °C, $P_{CO}$:$P_{O2}$:$P_{Ar}$=1:20:79, 60000 mL·$g_{cat}^{-1}$·$h^{-1}$, and 1.85 bar).

| Catalyst | $r_{100}'$ ($mmol_{CO}·mol_{Pt}^{-1}·s^{-1}$) | TOF'×$10^{-3}$ ($s^{-1}$) | $N_{CO}$ (μmol·$g_{cat}^{-1}$) | $N_{CO2}$ (μmol·$g_{cat}^{-1}$) | $\theta_{CO}$ | $\theta_{oxygen}$ | $lnA_0'$ |
|---|---|---|---|---|---|---|---|
| Pt/CNT-0 | 9.7 | 16.5 | 9.97 | 0.33 | 0.56 | 0.02 | 11.24 |
| Pt/CNT-200 | 19.9 | 30.4 | 10.59 | 0.96 | 0.54 | 0.05 | 10.21 |
| Pt/CNT-400 | 37.1 | 48.2 | 11.33 | 1.97 | 0.49 | 0.08 | 9.01 |
| Pt/CNT-600 | 39.0 | 53.8 | 9.77 | 2.64 | 0.45 | 0.12 | 8.27 |
| Pt/CNT-800 | 31.0 | 53.1 | 8.05 | 1.52 | 0.46 | 0.08 | 8.36 |
| Pt/CNT-1000 | 29.3 | 37.8 | 12.49 | 0.75 | 0.53 | 0.03 | 9.34 |

**Supplementary Table 8.** The kinetic parameters of a, b, and c for the nano-kinetics model using Pt charge and Pt B.E. as the kinetic descriptor, respectively. (100 $^{o}$C, $P_{CO}$:$P_{O2}$:$P_{Ar}$=1:20:79, 60000 mL·$g_{cat}^{-1}$·$h^{-1}$, and 1.85 bar).

| Parameters | Values | |
|---|---|---|
| | Pt charge | Pt B.E. |
| a | 2.81E-06 | 2.91E-158 |
| b | 7.200921 | 3.827937 |
| c | 2.443237 | 84.787753 |

**Supplementary Table 9.** The relationship between Pt particle size and the number of surface atoms.

| d | $N_T$ | m | $N_S$ | Dispersion ($N_S/N_T$) |
|---|---|---|---|---|
| 1.00 | 35.25218 | 1.966451 | 31.22859 | 0.885863 |
| 1.02 | 37.40989 | 1.992938 | 31.83202 | 0.850899 |
| 1.04 | 39.6539 | 2.019406 | 32.47705 | 0.819013 |
| 1.06 | 41.9859 | 2.045857 | 33.16364 | 0.789876 |
| 1.08 | 44.40759 | 2.07229 | 33.89174 | 0.763197 |
| 1.10 | 46.92065 | 2.098708 | 34.6613 | 0.738722 |
| 1.12 | 49.52677 | 2.125112 | 35.47226 | 0.716224 |
| 1.14 | 52.22765 | 2.151501 | 36.32459 | 0.695505 |
| 1.16 | 55.02498 | 2.177877 | 37.21825 | 0.676388 |
| 1.18 | 57.92045 | 2.20424 | 38.1532 | 0.658717 |
| 1.20 | 60.91576 | 2.230592 | 39.12939 | 0.642352 |
| 1.22 | 64.01259 | 2.256933 | 40.14682 | 0.627171 |
| 1.24 | 67.21264 | 2.283263 | 41.20543 | 0.613061 |
| 1.26 | 70.51761 | 2.309582 | 42.30521 | 0.599924 |
| 1.28 | 73.92917 | 2.335892 | 43.44612 | 0.587672 |
| 1.30 | 77.44903 | 2.362193 | 44.62816 | 0.576226 |
| 1.32 | 81.07887 | 2.388485 | 45.85128 | 0.565515 |
| 1.34 | 84.8204 | 2.414769 | 47.11547 | 0.555473 |
| 1.36 | 88.6753 | 2.441045 | 48.42072 | 0.546045 |
| 1.38 | 92.64525 | 2.467314 | 49.76699 | 0.537178 |
| 1.40 | 96.73197 | 2.493575 | 51.15427 | 0.528825 |
| 1.42 | 100.9371 | 2.519829 | 52.58256 | 0.520944 |
| 1.44 | 105.2624 | 2.546076 | 54.05182 | 0.513496 |
| 1.46 | 109.7096 | 2.572318 | 55.56205 | 0.506447 |
| 1.48 | 114.2802 | 2.598553 | 57.11323 | 0.499765 |
| 1.50 | 118.9761 | 2.624778 | 58.70509 | 0.493419 |
| 1.52 | 123.7989 | 2.651003 | 60.33822 | 0.487389 |
| 1.54 | 128.7503 | 2.677222 | 62.01224 | 0.481647 |
| 1.56 | 133.8319 | 2.703436 | 63.72715 | 0.476173 |
| 1.58 | 139.0456 | 2.729645 | 65.48293 | 0.470946 |
| 1.60 | 144.3929 | 2.755849 | 67.27959 | 0.465948 |
| 1.62 | 149.8756 | 2.782048 | 69.11712 | 0.461163 |
| 1.64 | 155.4954 | 2.808243 | 70.9955 | 0.456576 |
| 1.66 | 161.2539 | 2.834433 | 72.91474 | 0.452173 |
| 1.68 | 167.1528 | 2.860619 | 74.87483 | 0.447942 |
| 1.70 | 173.1939 | 2.886802 | 76.87575 | 0.443871 |
| 1.72 | 179.3789 | 2.91298 | 78.9175 | 0.439949 |
| 1.74 | 185.7093 | 2.939155 | 81.00008 | 0.436166 |
| 1.76 | 192.187 | 2.965324 | 83.12332 | 0.432513 |
| 1.78 | 198.8135 | 2.991493 | 85.28762 | 0.428983 |

| | | | | |
|---|---|---|---|---|
| 1.80 | 205.5907 | 3.017659 | 87.49268 | 0.425567 |
| 1.82 | 212.5201 | 3.04382 | 89.73851 | 0.422259 |
| 1.84 | 219.6036 | 3.069979 | 92.02512 | 0.419051 |
| 1.86 | 226.8427 | 3.096135 | 94.35257 | 0.415938 |
| 1.88 | 234.2391 | 3.122286 | 96.72068 | 0.412914 |
| 1.90 | 241.7947 | 3.148437 | 99.12967 | 0.409975 |
| 1.92 | 249.5109 | 3.174584 | 101.5794 | 0.407114 |
| 1.94 | 257.3897 | 3.200728 | 104.0699 | 0.404328 |
| 1.96 | 265.4325 | 3.226871 | 106.6013 | 0.401614 |
| 1.98 | 273.6412 | 3.253012 | 109.1734 | 0.398966 |
| 2.00 | 282.0174 | 3.279147 | 111.786 | 0.39638 |
| 2.02 | 290.5628 | 3.305282 | 114.4396 | 0.393855 |
| 2.04 | 299.2791 | 3.331415 | 117.1339 | 0.391387 |
| 2.06 | 308.168 | 3.357545 | 119.8689 | 0.388973 |
| 2.08 | 317.2312 | 3.383673 | 122.6447 | 0.38661 |
| 2.10 | 326.4704 | 3.4098 | 125.4613 | 0.384296 |
| 2.12 | 335.8872 | 3.435924 | 128.3185 | 0.382029 |
| 2.14 | 345.4834 | 3.462046 | 131.2165 | 0.379806 |
| 2.16 | 355.2607 | 3.488166 | 134.1552 | 0.377625 |
| 2.18 | 365.2207 | 3.514284 | 137.1346 | 0.375484 |
| 2.20 | 375.3652 | 3.540403 | 140.155 | 0.373383 |
| 2.22 | 385.6957 | 3.566519 | 143.2159 | 0.371318 |
| 2.24 | 396.2141 | 3.592633 | 146.3175 | 0.369289 |
| 2.26 | 406.9221 | 3.618745 | 149.4599 | 0.367294 |
| 2.28 | 417.8212 | 3.644855 | 152.643 | 0.365331 |
| 2.30 | 428.9132 | 3.670964 | 155.8668 | 0.363399 |
| 2.32 | 440.1998 | 3.697072 | 159.1313 | 0.361498 |
| 2.34 | 451.6827 | 3.723178 | 162.4365 | 0.359625 |
| 2.36 | 463.3636 | 3.749282 | 165.7824 | 0.35778 |
| 2.38 | 475.2442 | 3.775386 | 169.1691 | 0.355962 |
| 2.40 | 487.3261 | 3.801487 | 172.5964 | 0.35417 |
| 2.42 | 499.611 | 3.827588 | 176.0644 | 0.352403 |
| 2.44 | 512.1007 | 3.853687 | 179.5732 | 0.35066 |
| 2.46 | 524.7969 | 3.879785 | 183.1226 | 0.34894 |
| 2.48 | 537.7011 | 3.905881 | 186.7127 | 0.347243 |
| 2.50 | 550.8152 | 3.931977 | 190.3434 | 0.345567 |
| 2.52 | 564.1408 | 3.958073 | 194.0152 | 0.343913 |
| 2.54 | 577.6797 | 3.984166 | 197.7275 | 0.342279 |
| 2.56 | 591.4334 | 4.010259 | 201.4804 | 0.340665 |
| 2.58 | 605.4037 | 4.03635 | 205.274 | 0.33907 |
| 2.60 | 619.5922 | 4.06244 | 209.1084 | 0.337494 |
| 2.62 | 634.0008 | 4.088529 | 212.9834 | 0.335936 |
| 2.64 | 648.631 | 4.114618 | 216.8991 | 0.334395 |
| 2.66 | 663.4846 | 4.140705 | 220.8554 | 0.332872 |
| 2.68 | 678.5632 | 4.166791 | 224.8525 | 0.331366 |

| | | | | |
|---|---|---|---|---|
| 2.70 | 693.8686 | 4.192876 | 228.8902 | 0.329875 |
| 2.72 | 709.4024 | 4.218961 | 232.9687 | 0.328401 |
| 2.74 | 725.1663 | 4.245044 | 237.0878 | 0.326943 |
| 2.76 | 741.162 | 4.271127 | 241.2476 | 0.325499 |
| 2.78 | 757.3913 | 4.297209 | 245.4481 | 0.32407 |
| 2.80 | 773.8557 | 4.32329 | 249.6892 | 0.322656 |
| 2.82 | 790.5571 | 4.34937 | 253.9711 | 0.321256 |
| 2.84 | 807.497 | 4.375449 | 258.2936 | 0.319869 |
| 2.86 | 824.6773 | 4.401528 | 262.6568 | 0.318496 |
| 2.88 | 842.0994 | 4.427606 | 267.0606 | 0.317137 |
| 2.90 | 859.7653 | 4.453683 | 271.5052 | 0.31579 |
| 2.92 | 877.6765 | 4.479759 | 275.9904 | 0.314456 |
| 2.94 | 895.8348 | 4.505835 | 280.5163 | 0.313134 |
| 2.96 | 914.2418 | 4.53191 | 285.0828 | 0.311824 |
| 2.98 | 932.8992 | 4.557984 | 289.6901 | 0.310527 |
| 3.00 | 951.8087 | 4.584058 | 294.338 | 0.309241 |
| 3.02 | 970.9721 | 4.61013 | 299.0266 | 0.307966 |
| 3.04 | 990.391 | 4.636203 | 303.7558 | 0.306703 |
| 3.06 | 1010.067 | 4.662274 | 308.5257 | 0.305451 |
| 3.08 | 1030.002 | 4.688345 | 313.3363 | 0.304209 |
| 3.10 | 1050.198 | 4.714416 | 318.1876 | 0.302979 |
| 3.12 | 1070.655 | 4.740485 | 323.0795 | 0.301759 |
| 3.14 | 1091.377 | 4.766555 | 328.0121 | 0.300549 |
| 3.16 | 1112.365 | 4.792623 | 332.9853 | 0.299349 |
| 3.18 | 1133.619 | 4.818691 | 337.9992 | 0.298159 |
| 3.20 | 1155.143 | 4.844759 | 343.0538 | 0.29698 |
| 3.22 | 1176.938 | 4.870826 | 348.1491 | 0.295809 |
| 3.24 | 1199.005 | 4.896892 | 353.285 | 0.294648 |
| 3.26 | 1221.346 | 4.922958 | 358.4616 | 0.293497 |
| 3.28 | 1243.963 | 4.949024 | 363.6788 | 0.292355 |
| 3.30 | 1266.857 | 4.975089 | 368.9367 | 0.291222 |
| 3.32 | 1290.031 | 5.001153 | 374.2353 | 0.290098 |
| 3.34 | 1313.486 | 5.027217 | 379.5745 | 0.288983 |
| 3.36 | 1337.223 | 5.05328 | 384.9544 | 0.287876 |
| 3.38 | 1361.244 | 5.079343 | 390.3749 | 0.286778 |
| 3.40 | 1385.551 | 5.105406 | 395.8361 | 0.285689 |
| 3.42 | 1410.147 | 5.131468 | 401.338 | 0.284607 |
| 3.44 | 1435.031 | 5.15753 | 406.8805 | 0.283534 |
| 3.46 | 1460.206 | 5.183591 | 412.4637 | 0.28247 |
| 3.48 | 1485.674 | 5.209652 | 418.0875 | 0.281413 |
| 3.50 | 1511.437 | 5.235712 | 423.7521 | 0.280364 |
| 3.52 | 1537.496 | 5.261772 | 429.4572 | 0.279322 |
| 3.54 | 1563.852 | 5.287831 | 435.203 | 0.278289 |
| 3.56 | 1590.508 | 5.313891 | 440.9895 | 0.277263 |
| 3.58 | 1617.465 | 5.339949 | 446.8164 | 0.276245 |

| | | | | |
|---|---|---|---|---|
| 3.60 | 1644.725 | 5.366007 | 452.6843 | 0.275234 |
| 3.62 | 1672.29 | 5.392065 | 458.5927 | 0.27423 |
| 3.64 | 1700.161 | 5.418123 | 464.5419 | 0.273234 |
| 3.66 | 1728.34 | 5.44418 | 470.5316 | 0.272245 |
| 3.68 | 1756.829 | 5.470237 | 476.5621 | 0.271263 |
| 3.70 | 1785.628 | 5.496294 | 482.6332 | 0.270288 |
| 3.72 | 1814.741 | 5.52235 | 488.7449 | 0.269319 |
| 3.74 | 1844.169 | 5.548406 | 494.8973 | 0.268358 |
| 3.76 | 1873.913 | 5.574461 | 501.0903 | 0.267403 |
| 3.78 | 1903.975 | 5.600517 | 507.324 | 0.266455 |
| 3.80 | 1934.357 | 5.626572 | 513.5984 | 0.265514 |
| 3.82 | 1965.061 | 5.652626 | 519.9133 | 0.264579 |
| 3.84 | 1996.088 | 5.67868 | 526.269 | 0.26365 |
| 3.86 | 2027.439 | 5.704734 | 532.6653 | 0.262728 |
| 3.88 | 2059.117 | 5.730788 | 539.1022 | 0.261812 |
| 3.90 | 2091.124 | 5.756841 | 545.5799 | 0.260903 |
| 3.92 | 2123.46 | 5.782894 | 552.0981 | 0.259999 |
| 3.94 | 2156.128 | 5.808947 | 558.657 | 0.259102 |
| 3.96 | 2189.13 | 5.834999 | 565.2566 | 0.258211 |
| 3.98 | 2222.466 | 5.861051 | 571.8968 | 0.257325 |
| 4.00 | 2256.139 | 5.887103 | 578.5776 | 0.256446 |
| 4.02 | 2290.151 | 5.913155 | 585.2992 | 0.255572 |
| 4.04 | 2324.502 | 5.939206 | 592.0613 | 0.254705 |
| 4.06 | 2359.196 | 5.965257 | 598.8641 | 0.253842 |
| 4.08 | 2394.233 | 5.991308 | 605.7076 | 0.252986 |
| 4.10 | 2429.615 | 6.017359 | 612.5917 | 0.252135 |
| 4.12 | 2465.344 | 6.043409 | 619.5165 | 0.25129 |
| 4.14 | 2501.422 | 6.069458 | 626.4818 | 0.25045 |
| 4.16 | 2537.85 | 6.095508 | 633.4879 | 0.249616 |
| 4.18 | 2574.63 | 6.121558 | 640.5346 | 0.248787 |
| 4.20 | 2611.763 | 6.147607 | 647.622 | 0.247964 |
| 4.22 | 2649.252 | 6.173657 | 654.75 | 0.247145 |
| 4.24 | 2687.098 | 6.199705 | 661.9187 | 0.246332 |
| 4.26 | 2725.303 | 6.225754 | 669.128 | 0.245524 |
| 4.28 | 2763.868 | 6.251802 | 676.378 | 0.244722 |
| 4.30 | 2802.795 | 6.27785 | 683.6686 | 0.243924 |
| 4.32 | 2842.086 | 6.303898 | 690.9998 | 0.243131 |
| 4.34 | 2881.742 | 6.329946 | 698.3717 | 0.242344 |
| 4.36 | 2921.766 | 6.355994 | 705.7843 | 0.241561 |
| 4.38 | 2962.158 | 6.382041 | 713.2375 | 0.240783 |
| 4.40 | 3002.921 | 6.408088 | 720.7313 | 0.24001 |
| 4.42 | 3044.057 | 6.434135 | 728.2658 | 0.239242 |
| 4.44 | 3085.566 | 6.460182 | 735.8409 | 0.238478 |
| 4.46 | 3127.451 | 6.486228 | 743.4567 | 0.23772 |
| 4.48 | 3169.713 | 6.512274 | 751.1132 | 0.236966 |

| | | | | |
|---|---|---|---|---|
| 4.50 | 3212.354 | 6.538321 | 758.8103 | 0.236216 |
| 4.52 | 3255.377 | 6.564366 | 766.548 | 0.235471 |
| 4.54 | 3298.781 | 6.590412 | 774.3263 | 0.234731 |
| 4.56 | 3342.57 | 6.616457 | 782.1453 | 0.233995 |
| 4.58 | 3386.744 | 6.642503 | 790.005 | 0.233264 |
| 4.60 | 3431.306 | 6.668548 | 797.9053 | 0.232537 |
| 4.62 | 3476.257 | 6.694593 | 805.8463 | 0.231814 |
| 4.64 | 3521.599 | 6.720638 | 813.8279 | 0.231096 |
| 4.66 | 3567.333 | 6.746682 | 821.8501 | 0.230382 |
| 4.68 | 3613.462 | 6.772727 | 829.913 | 0.229673 |
| 4.70 | 3659.987 | 6.798771 | 838.0166 | 0.228967 |
| 4.72 | 3706.909 | 6.824815 | 846.1608 | 0.228266 |
| 4.74 | 3754.231 | 6.850859 | 854.3456 | 0.227569 |
| 4.76 | 3801.953 | 6.876903 | 862.5711 | 0.226876 |
| 4.78 | 3850.079 | 6.902946 | 870.8372 | 0.226187 |
| 4.80 | 3898.609 | 6.92899 | 879.1439 | 0.225502 |
| 4.82 | 3947.545 | 6.955033 | 887.4913 | 0.224821 |
| 4.84 | 3996.888 | 6.981076 | 895.8794 | 0.224144 |
| 4.86 | 4046.641 | 7.007119 | 904.3081 | 0.223471 |
| 4.88 | 4096.806 | 7.033162 | 912.7774 | 0.222802 |
| 4.90 | 4147.383 | 7.059204 | 921.2874 | 0.222137 |
| 4.92 | 4198.375 | 7.085247 | 929.838 | 0.221476 |
| 4.94 | 4249.783 | 7.11129 | 938.4294 | 0.220818 |
| 4.96 | 4301.609 | 7.137332 | 947.0613 | 0.220164 |
| 4.98 | 4353.855 | 7.163374 | 955.7339 | 0.219514 |
| 5.00 | 4406.522 | 7.189416 | 964.4471 | 0.218868 |

**Supplementary Table 10.** The kinetics modeling of catalytic activity based on Pt binding energy (row) and Pt particle size (column). (100 ºC, $P_{CO}$:$P_{O2}$:$P_{Ar}$=1:20:79, 60000 mL·$g_{cat}^{-1}$·$h^{-1}$, and 1.85 bar).

| | 71.56 | 71.60 | 71.65 | 71.70 | 71.75 | 71.80 | 71.85 | 71.90 | 71.95 | 72.00 | 72.05 |
|---|---|---|---|---|---|---|---|---|---|---|---|
| 1.00 | 15.84 | 18.46 | 22.35 | 27.07 | 32.78 | 39.69 | 48.07 | 58.20 | 70.48 | 85.35 | 103.35 |
| 1.10 | 13.21 | 15.39 | 18.64 | 22.57 | 27.33 | 33.10 | 40.08 | 48.54 | 58.77 | 71.17 | 86.19 |
| 1.20 | 11.48 | 13.39 | 16.21 | 19.63 | 23.77 | 28.78 | 34.85 | 42.20 | 51.11 | 61.89 | 74.94 |
| 1.30 | 10.30 | 12.01 | 14.54 | 17.61 | 21.32 | 25.82 | 31.26 | 37.86 | 45.85 | 55.52 | 67.23 |
| 1.40 | 9.46 | 11.02 | 13.34 | 16.16 | 19.57 | 23.69 | 28.69 | 34.75 | 42.07 | 50.95 | 61.70 |
| 1.50 | 8.82 | 10.28 | 12.45 | 15.08 | 18.26 | 22.11 | 26.77 | 32.42 | 39.26 | 47.54 | 57.57 |
| 1.60 | 8.33 | 9.71 | 11.76 | 14.24 | 17.24 | 20.88 | 25.28 | 30.61 | 37.07 | 44.89 | 54.36 |
| 1.70 | 7.94 | 9.25 | 11.20 | 13.56 | 16.42 | 19.89 | 24.08 | 29.16 | 35.32 | 42.76 | 51.79 |
| 1.80 | 7.61 | 8.87 | 10.74 | 13.00 | 15.75 | 19.07 | 23.09 | 27.96 | 33.86 | 41.00 | 49.65 |
| 1.90 | 7.33 | 8.54 | 10.34 | 12.53 | 15.17 | 18.37 | 22.24 | 26.94 | 32.62 | 39.50 | 47.83 |
| 2.00 | 7.09 | 8.26 | 10.00 | 12.11 | 14.67 | 17.76 | 21.51 | 26.04 | 31.54 | 38.19 | 46.25 |
| 2.10 | 6.87 | 8.01 | 9.70 | 11.74 | 14.22 | 17.22 | 20.85 | 25.25 | 30.58 | 37.03 | 44.84 |
| 2.20 | 6.68 | 7.78 | 9.42 | 11.41 | 13.82 | 16.73 | 20.26 | 24.53 | 29.71 | 35.97 | 43.56 |
| 2.30 | 6.50 | 7.57 | 9.17 | 11.10 | 13.45 | 16.28 | 19.72 | 23.88 | 28.91 | 35.01 | 42.40 |
| 2.40 | 6.33 | 7.38 | 8.94 | 10.82 | 13.10 | 15.87 | 19.22 | 23.27 | 28.18 | 34.12 | 41.32 |
| 2.50 | 6.18 | 7.20 | 8.72 | 10.56 | 12.79 | 15.48 | 18.75 | 22.70 | 27.49 | 33.29 | 40.32 |
| 2.60 | 6.03 | 7.03 | 8.52 | 10.31 | 12.49 | 15.12 | 18.31 | 22.17 | 26.85 | 32.52 | 39.37 |
| 2.70 | 5.90 | 6.87 | 8.32 | 10.08 | 12.21 | 14.78 | 17.90 | 21.67 | 26.25 | 31.78 | 38.49 |
| 2.80 | 5.77 | 6.72 | 8.14 | 9.86 | 11.94 | 14.46 | 17.51 | 21.20 | 25.67 | 31.09 | 37.64 |
| 2.90 | 5.65 | 6.58 | 7.97 | 9.65 | 11.68 | 14.15 | 17.13 | 20.75 | 25.13 | 30.42 | 36.84 |
| 3.00 | 5.53 | 6.44 | 7.80 | 9.45 | 11.44 | 13.86 | 16.78 | 20.32 | 24.60 | 29.79 | 36.08 |
| 3.10 | 5.42 | 6.31 | 7.65 | 9.26 | 11.21 | 13.58 | 16.44 | 19.91 | 24.11 | 29.19 | 35.35 |
| 3.20 | 5.31 | 6.19 | 7.49 | 9.07 | 10.99 | 13.31 | 16.11 | 19.51 | 23.63 | 28.61 | 34.65 |
| 3.30 | 5.21 | 6.07 | 7.35 | 8.90 | 10.78 | 13.05 | 15.80 | 19.13 | 23.17 | 28.06 | 33.98 |
| 3.40 | 5.11 | 5.95 | 7.21 | 8.73 | 10.57 | 12.80 | 15.50 | 18.77 | 22.73 | 27.52 | 33.33 |
| 3.50 | 5.01 | 5.84 | 7.07 | 8.57 | 10.37 | 12.56 | 15.21 | 18.42 | 22.31 | 27.01 | 32.71 |
| 3.60 | 4.92 | 5.74 | 6.95 | 8.41 | 10.18 | 12.33 | 14.93 | 18.08 | 21.90 | 26.52 | 32.11 |
| 3.70 | 4.83 | 5.63 | 6.82 | 8.26 | 10.00 | 12.11 | 14.67 | 17.76 | 21.50 | 26.04 | 31.53 |
| 3.80 | 4.75 | 5.53 | 6.70 | 8.11 | 9.82 | 11.90 | 14.41 | 17.45 | 21.12 | 25.58 | 30.98 |
| 3.90 | 4.66 | 5.44 | 6.58 | 7.97 | 9.65 | 11.69 | 14.16 | 17.14 | 20.76 | 25.14 | 30.44 |
| 4.00 | 4.59 | 5.34 | 6.47 | 7.84 | 9.49 | 11.49 | 13.91 | 16.85 | 20.40 | 24.71 | 29.92 |
| 4.10 | 4.51 | 5.25 | 6.36 | 7.70 | 9.33 | 11.30 | 13.68 | 16.57 | 20.06 | 24.29 | 29.42 |
| 4.20 | 4.43 | 5.17 | 6.26 | 7.58 | 9.17 | 11.11 | 13.45 | 16.29 | 19.73 | 23.89 | 28.93 |
| 4.30 | 4.36 | 5.08 | 6.15 | 7.45 | 9.03 | 10.93 | 13.23 | 16.03 | 19.41 | 23.50 | 28.46 |
| 4.40 | 4.29 | 5.00 | 6.06 | 7.33 | 8.88 | 10.75 | 13.02 | 15.77 | 19.10 | 23.12 | 28.00 |
| 4.50 | 4.22 | 4.92 | 5.96 | 7.22 | 8.74 | 10.58 | 12.82 | 15.52 | 18.79 | 22.76 | 27.56 |
| 4.60 | 4.16 | 4.85 | 5.87 | 7.11 | 8.60 | 10.42 | 12.62 | 15.28 | 18.50 | 22.40 | 27.13 |
| 4.70 | 4.09 | 4.77 | 5.78 | 7.00 | 8.47 | 10.26 | 12.42 | 15.04 | 18.22 | 22.06 | 26.71 |
| 4.80 | 4.03 | 4.70 | 5.69 | 6.89 | 8.34 | 10.10 | 12.24 | 14.82 | 17.94 | 21.73 | 26.31 |
| 4.90 | 3.97 | 4.63 | 5.61 | 6.79 | 8.22 | 9.95 | 12.05 | 14.60 | 17.67 | 21.40 | 25.92 |
| 5.00 | 3.91 | 4.56 | 5.52 | 6.69 | 8.10 | 9.81 | 11.88 | 14.38 | 17.41 | 21.09 | 25.53 |

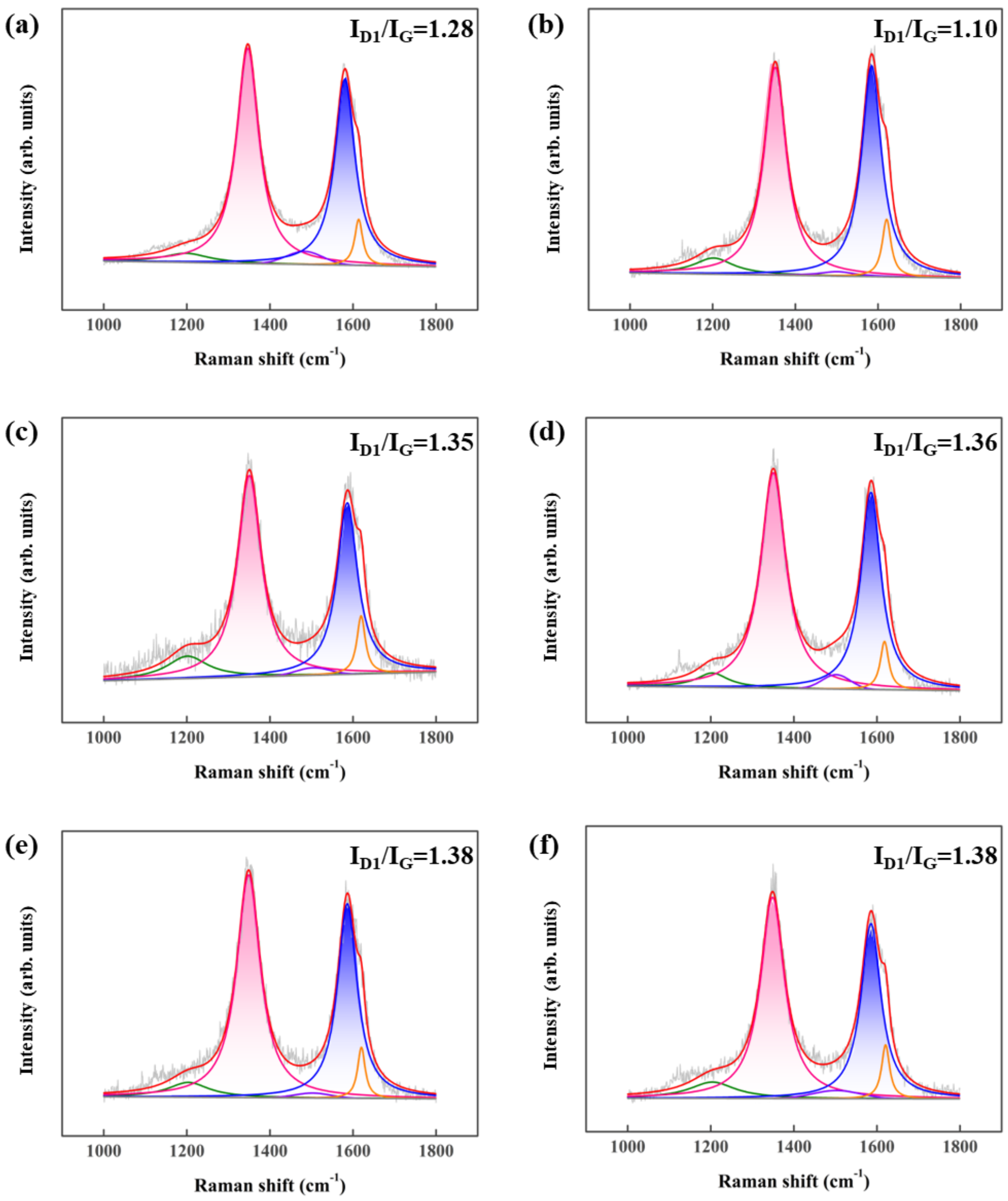


**Supplementary Figure 1.** Raman spectra of (a) CNT-0, (b) CNT-200, (c) CNT-400, (d) CNT-600, (e) CNT-800, and (f) CNT-1000.

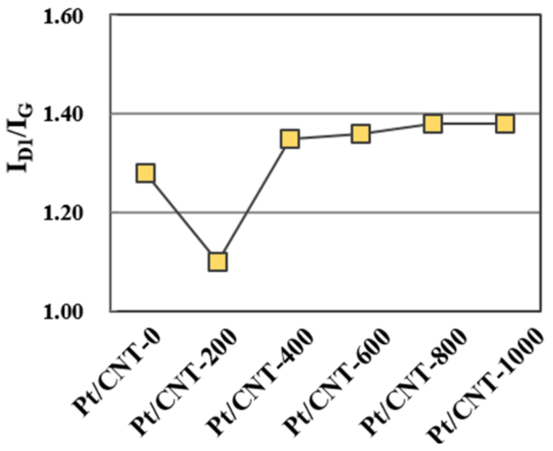


**Supplementary Figure 2.** The intensity ratio of D1 band to G band for CNT-0, CNT-200, CNT-400, CNT-600, CNT-800, and CNT-1000.

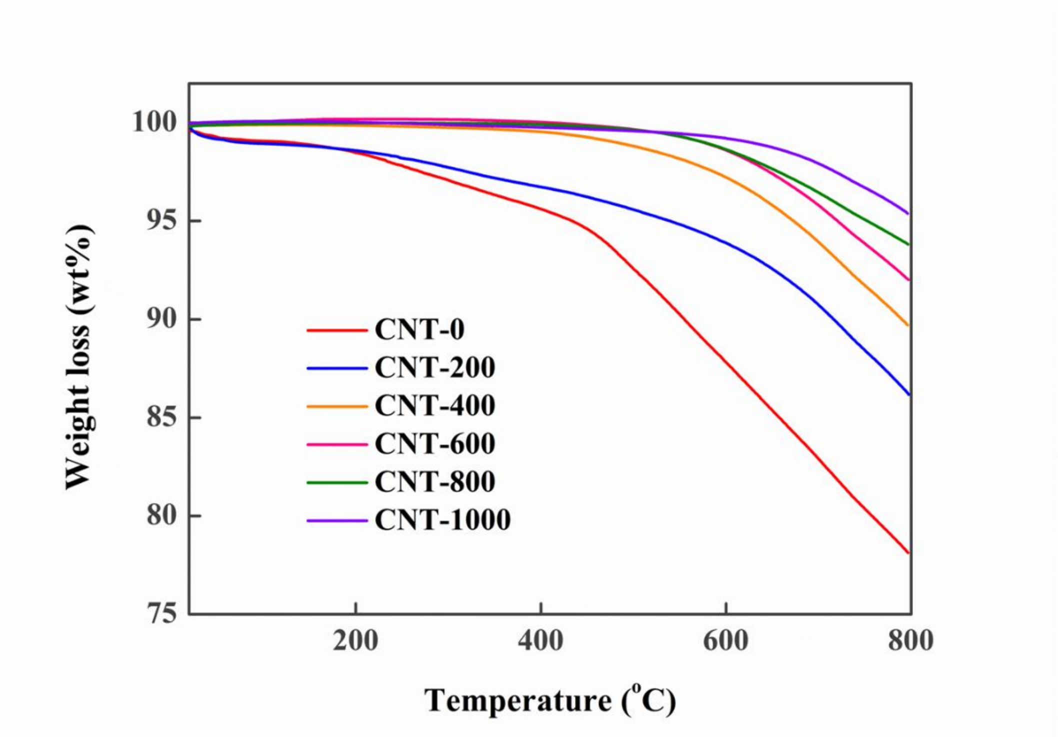


**Supplementary Figure 3.** TG profiles of CNT-0, CNT-200, CNT-400, CNT-600, CNT-800 and CNT-1000.

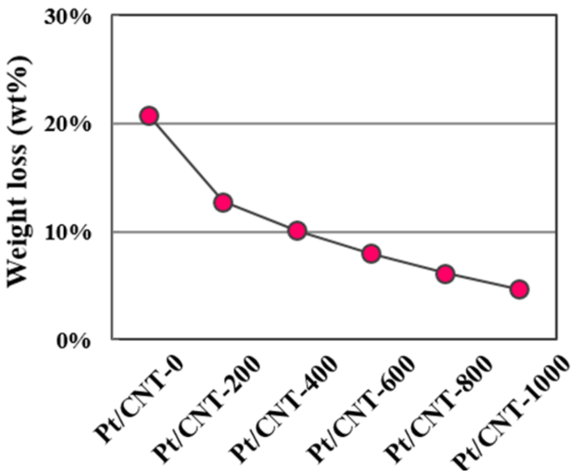


**Supplementary Figure 4.** The weight loss based on the TG profiles of CNT-0, CNT-200, CNT-400, CNT-600, CNT-800 and CNT-1000.

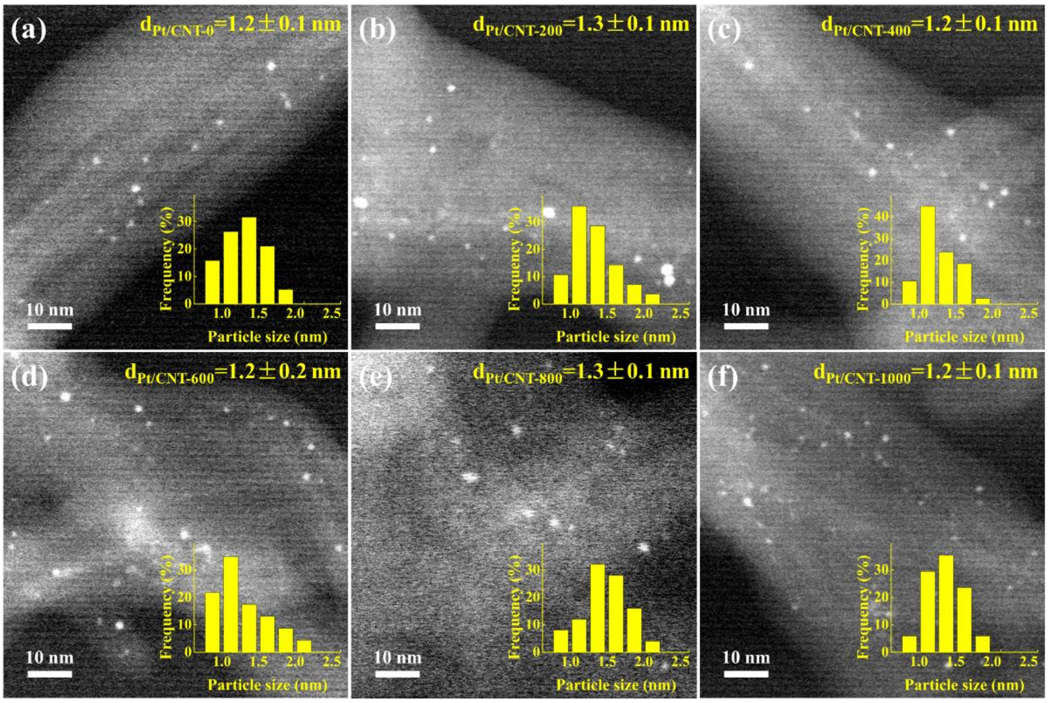


**Supplementary Figure 5.** Typical HAADF-STEM images of (a) Pt/CNT-0, (b) Pt/CNT-200, (c) Pt/CNT-400, (d) Pt/CNT-600, (e) Pt/CNT-800 and (f) Pt/CNT-1000.

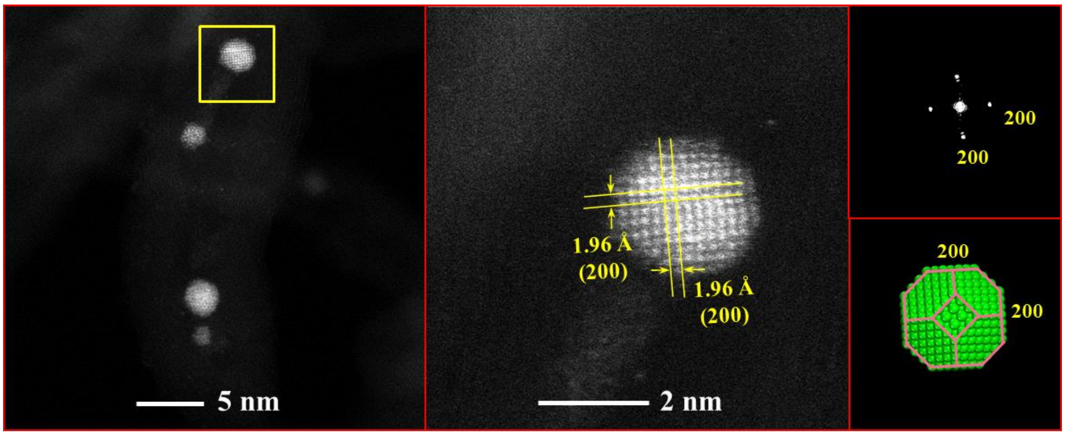


**Supplementary Figure 6.** Typical Aberration-corrected HAADF-STEM, the corresponding FFT pattern and schematic diagram of a truncated octahedron model of Pt/CNT-0.

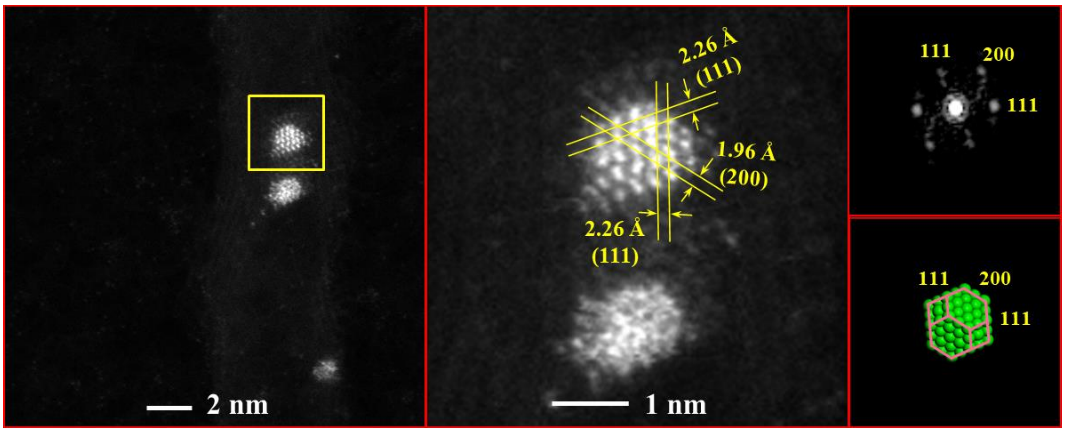


**Supplementary Figure 7.** Typical Aberration-corrected HAADF-STEM, the corresponding FFT pattern and schematic diagram of a truncated octahedron model of Pt/CNT-1000.

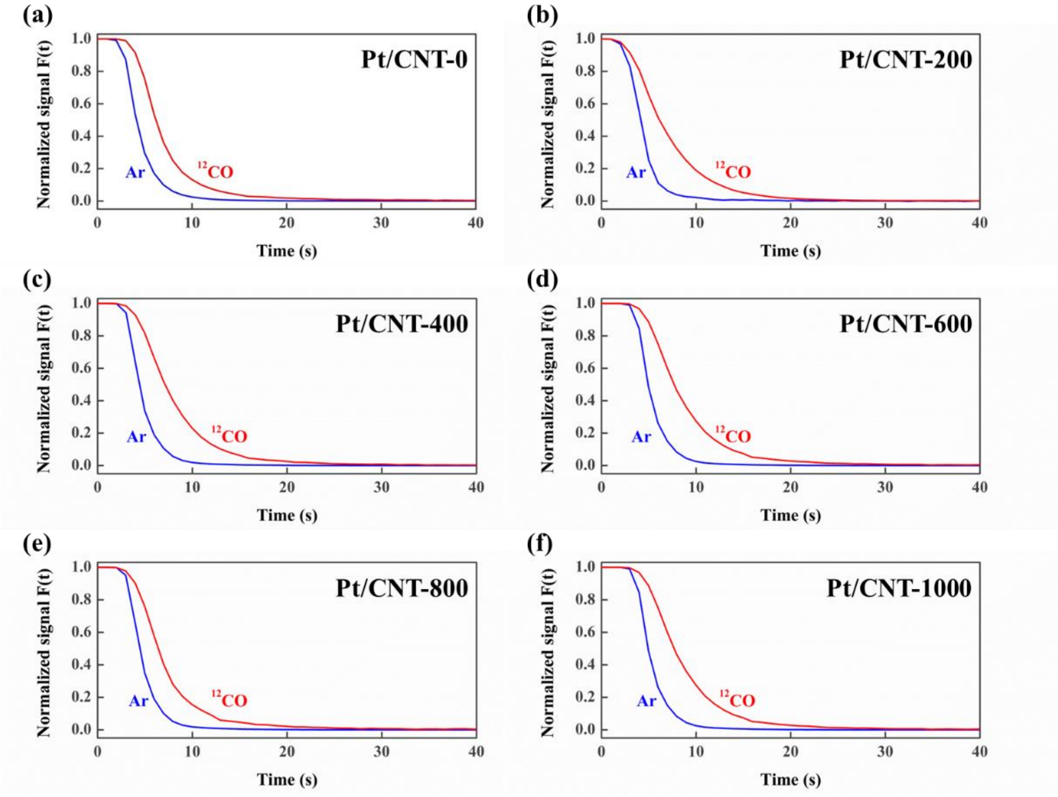


**Supplementary Figure 8.** The steady-state isotopic transients of Ar and $^{12}C^{16}O$ following a switch from Ar/$^{12}C^{16}O$ to Kr/$^{13}C^{16}O$ for (a) Pt/CNT-0, (b) Pt/CNT-200, (c) Pt/CNT-400, (d) Pt/CNT-600, (e) Pt/CNT-800 and (f) Pt/CNT-1000. (100 ºC, $P_{CO}$:$P_{Ar}$=1:99, 60000 mL·$g_{cat}^{-1}$·$h^{-1}$, and 1.85 bar).

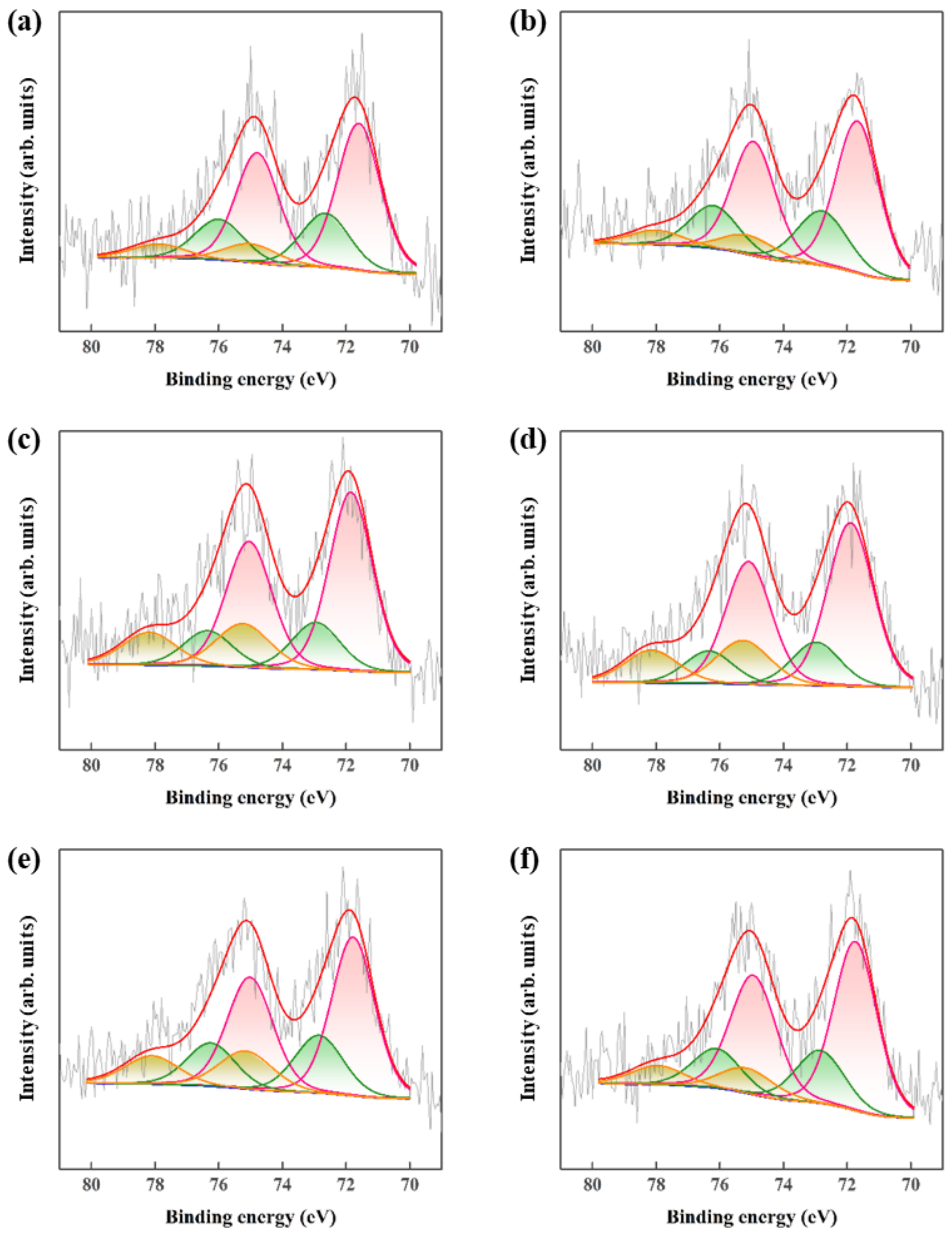


**Supplementary Figure 9.** Typical XPS Pt *4f* spectra of (a) Pt/CNT-0, (b) Pt/CNT-200, (c) Pt/CNT-400, (d) Pt/CNT-600, (e) Pt/CNT-800 and (f) Pt/CNT-1000.

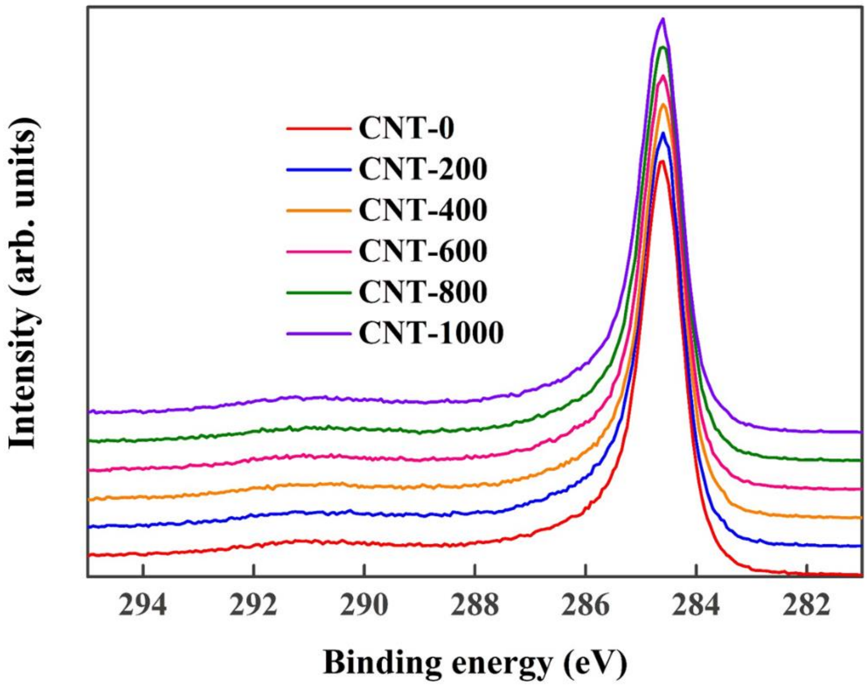


**Supplementary Figure 10.** Typical XPS C *1s* spectra of Pt/CNT-0, Pt/CNT-200, Pt/CNT-400, Pt/CNT-600, Pt/CNT-800, and Pt/CNT-1000.

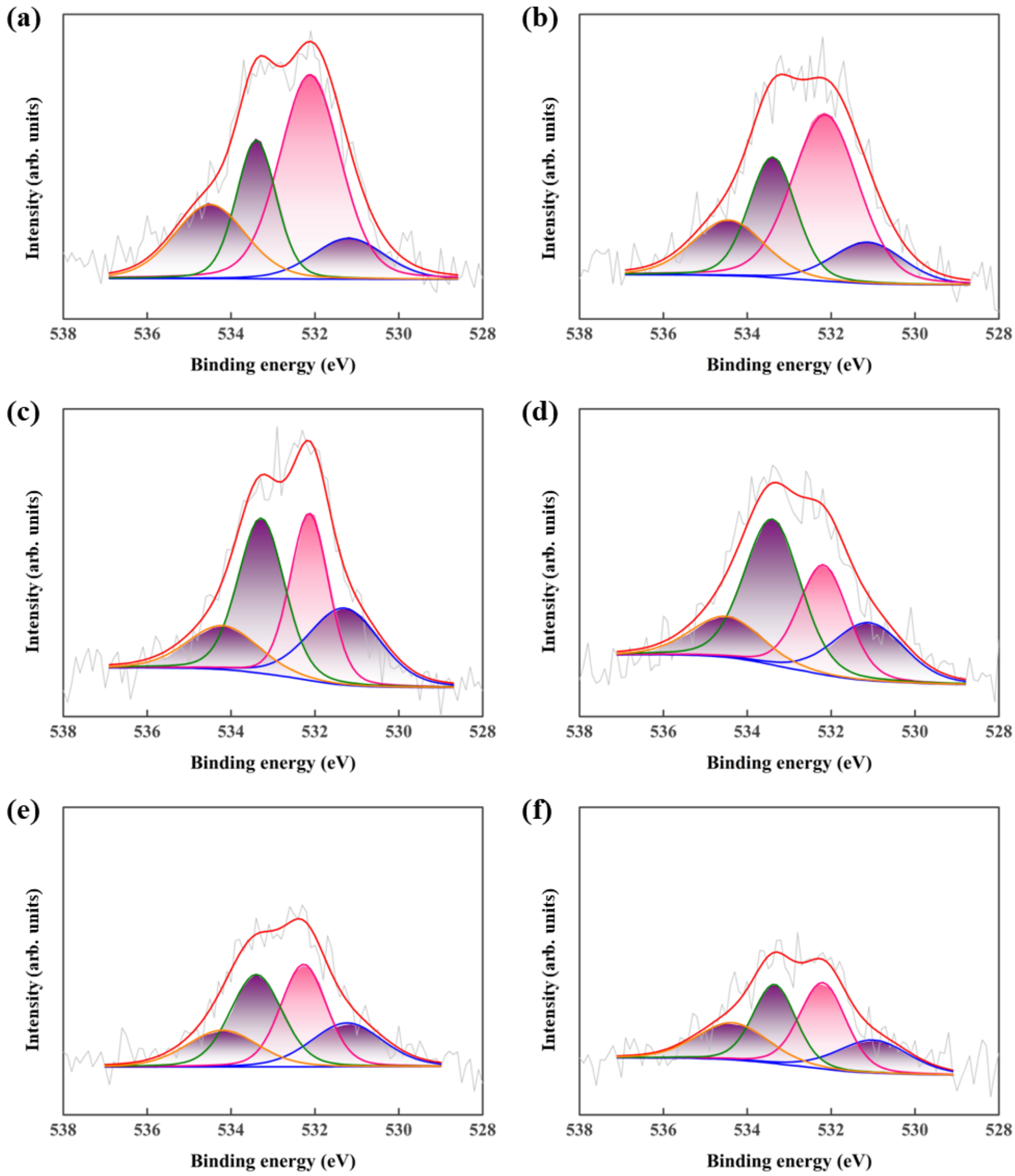


**Supplementary Figure 11.** Typical XPS O *1s* spectra of (a) Pt/CNT-0, (b) Pt/CNT-200, (c) Pt/CNT-400, (d) Pt/CNT-600, (e) Pt/CNT-800 and (f) Pt/CNT-1000.

$$CO + * \underset{}{\overset{K_1'}{\rightleftharpoons}} CO* \quad (1.1)$$

$$O_2 + * \overset{K_2'}{\rightleftharpoons} O_2* \quad (1.2)$$

$$CO* + O_2* \xrightarrow{k_3'} CO_2* + O* \quad (1.3)$$

$$CO* + O* \xrightarrow{k_4'} CO_2* + * \quad (1.4)$$

$$CO_2* \xrightarrow{k_5'} CO_2 + * \quad (1.5)$$

**Supplementary Figure 12.** Proposed pathway of CO-assisted $O_2$ dissociation via the formation of OOCO for CO oxidation.

$$CO + * \underset{}{\overset{K_1}{\rightleftharpoons}} CO* \qquad (2.1)$$

$$O_2 + 2* \xrightarrow{k_2} O_2* \qquad (2.2)$$

$$O_2* + * \xrightarrow{k_3} 2O* \qquad (2.3)$$

$$CO* + O* \xrightarrow{k_4} CO_2* + * \qquad (2.4)$$

$$CO_2* \xrightarrow{k_5} CO_2 + * \qquad (2.5)$$

**Supplementary Figure 13.** Proposed pathway of direct $O_2$ dissociation for CO oxidation.

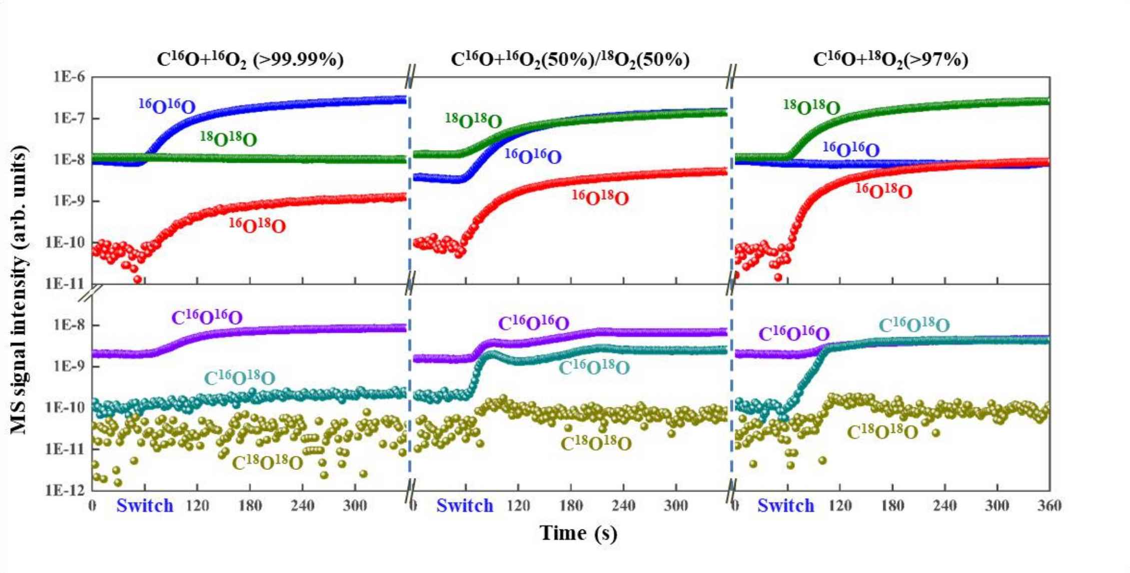


**Supplementary Figure 14.** Mass spectrometry (MS) data collected for the Pt/CNT-0 catalyst during the switch from Ar to Ar+$C^{16}O+^{16}O_2$ (>99.9%), Ar+$C^{16}O+^{16}O_2$ (50%)/$^{18}O_2$ (50%), and Ar+$C^{16}O+^{18}O_2$ (>97%). Reaction conditions: 100 ºC, ambient pressure, $P_{CO}:P_{O2}:P_{Ar}$=1:20:79, 60000 mL·$g_{cat}^{-1}$·$h^{-1}$.

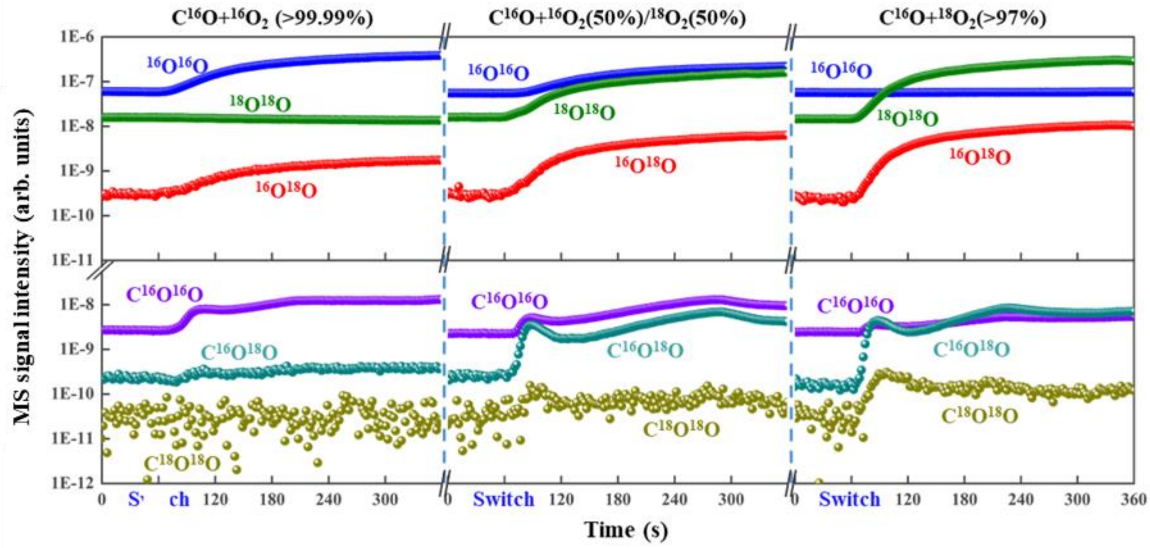


**Supplementary Figure 15.** Mass spectrometry (MS) data collected for the Pt/CNT-200 catalyst during the switch from Ar to Ar+$C^{16}O$+$^{16}O_2$ (>99.9%), Ar+$C^{16}O$+$^{16}O_2$ (50%)/$^{18}O_2$ (50%), and Ar+$C^{16}O$+$^{18}O_2$ (>97%). Reaction conditions: 100 °C, ambient pressure, $P_{CO}$:$P_{O2}$:$P_{Ar}$=1:20:79, 60000 mL·$g_{cat}^{-1}$·$h^{-1}$.

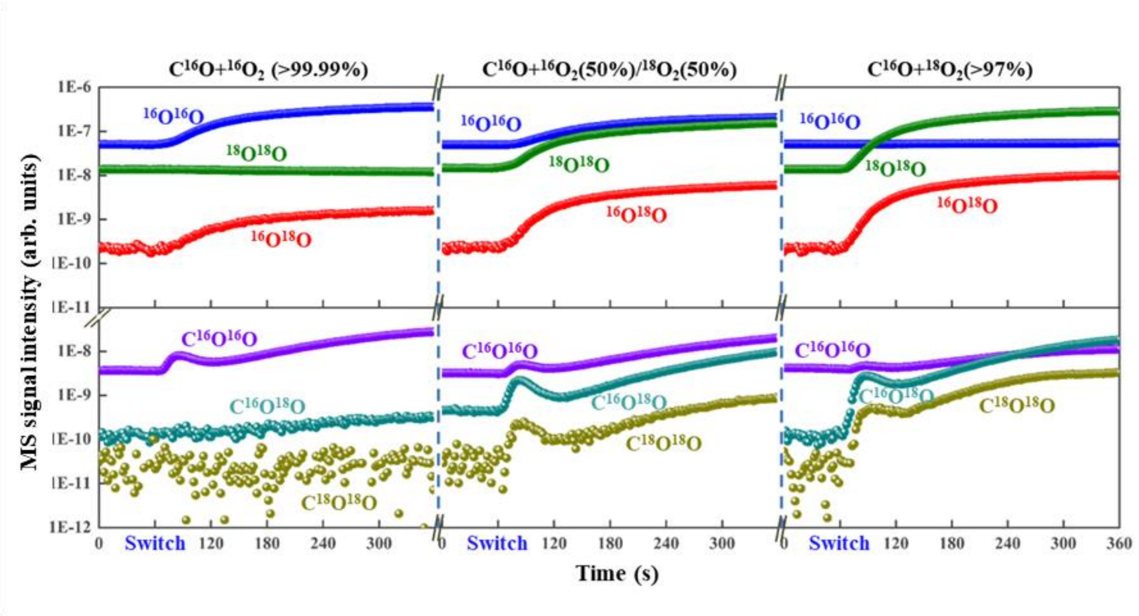


**Supplementary Figure 16.** Mass spectrometry (MS) data collected for the Pt/CNT-800 catalyst during the switch from Ar to Ar+$C^{16}O$+$^{16}O_2$ (>99.9%), Ar+$C^{16}O$+$^{16}O_2$ (50%)/$^{18}O_2$ (50%), and Ar+$C^{16}O$+$^{18}O_2$ (>97%). Reaction conditions: 100 °C, ambient pressure, $P_{CO}$:$P_{O2}$:$P_{Ar}$=1:20:79, 60000 mL·$g_{cat}^{-1}$·$h^{-1}$.

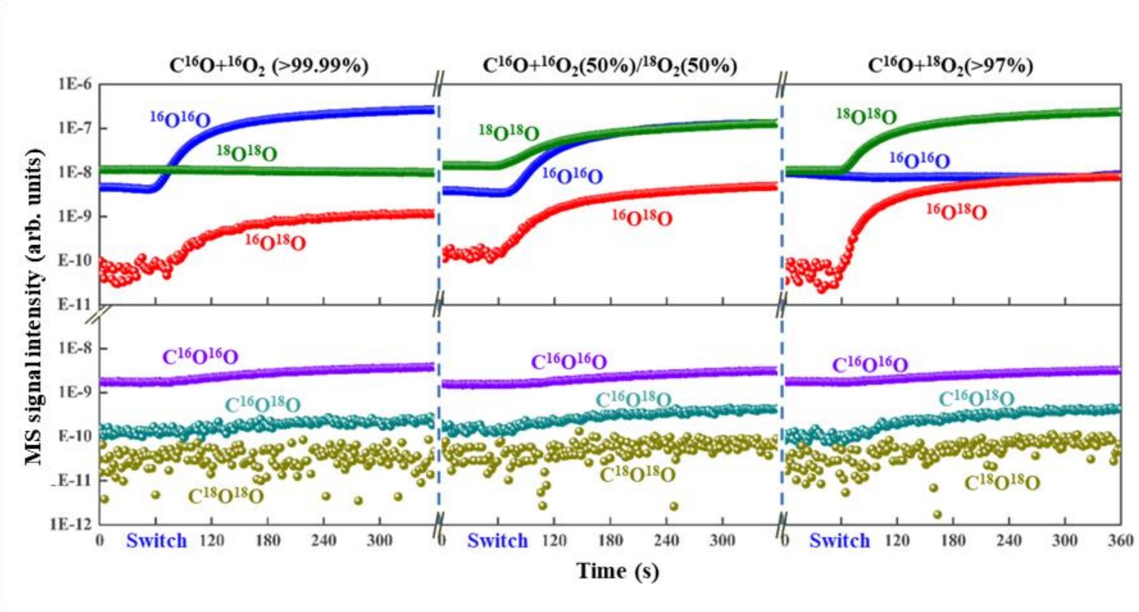


**Supplementary Figure 17.** Mass spectrometry (MS) data collected without catalyst (blank) during the switch from Ar to Ar+C$^{16}$O+$^{16}O_2$ (>99.9%), Ar+C$^{16}$O+$^{16}O_2$ (50%)/$^{18}O_2$ (50%), and Ar+C$^{16}$O+$^{18}O_2$ (>97%). Reaction conditions: 100 ºC, ambient pressure, $P_{CO}$:$P_{O2}$:$P_{Ar}$=1:20:79, 60000 mL·$g_{cat}^{-1}$·$h^{-1}$.

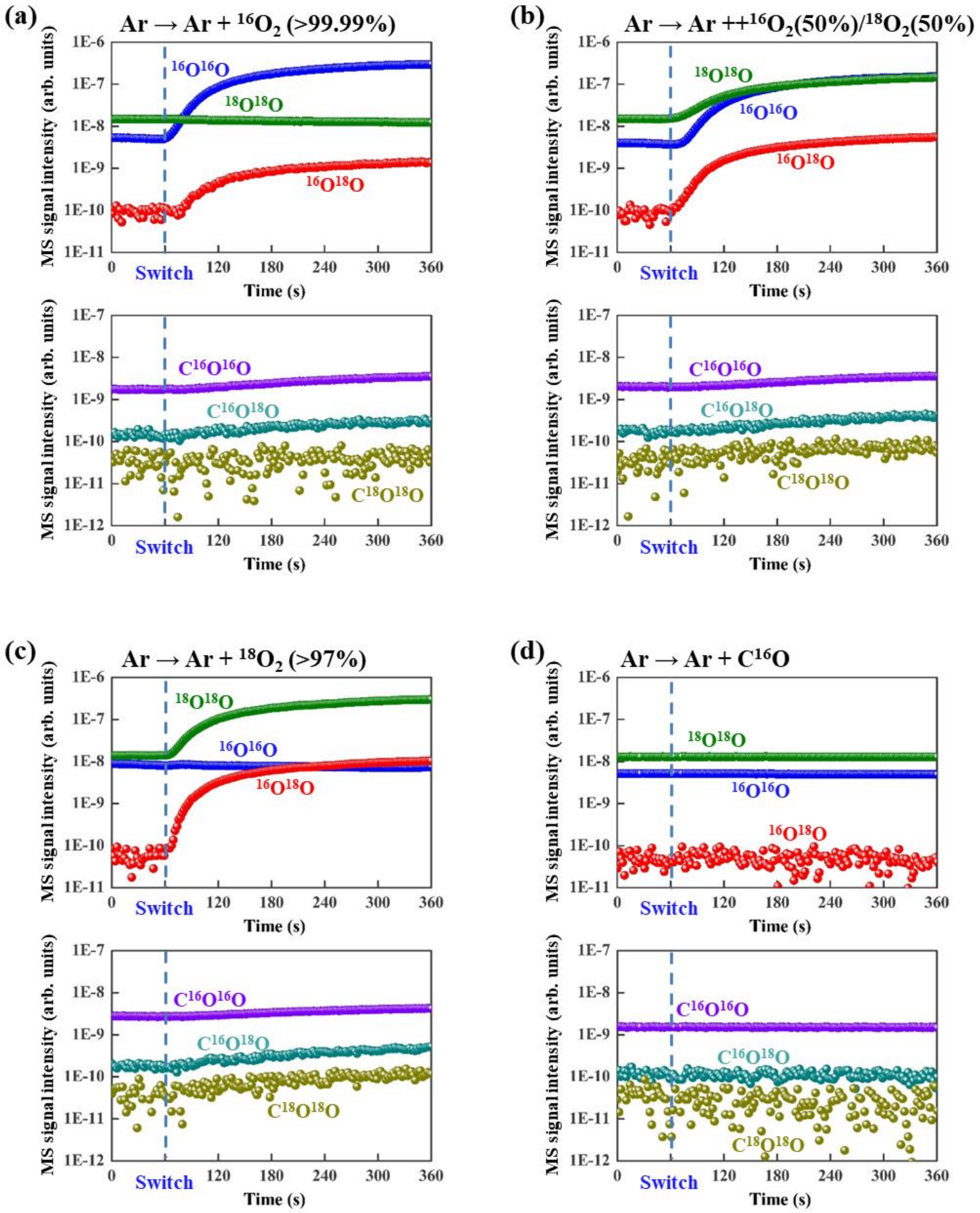


**Supplementary Figure 18.** Mass spectrometry (MS) data collected without catalyst (blank) during the switch from Ar to (a) Ar+$^{16}O_2$ (>99.9%), (b) Ar+$^{16}O_2$ (50%)/$^{18}O_2$ (50%), (c) Ar+$^{18}O_2$ (>97%) and (d) Ar+$C^{16}O$. Reaction conditions: 100 ºC, ambient pressure, $P_{O2}$:$P_{Ar}$=20:80, $P_{CO}$:$P_{Ar}$=1:99, 60000 mL·$g_{cat}^{-1}$·$h^{-1}$.

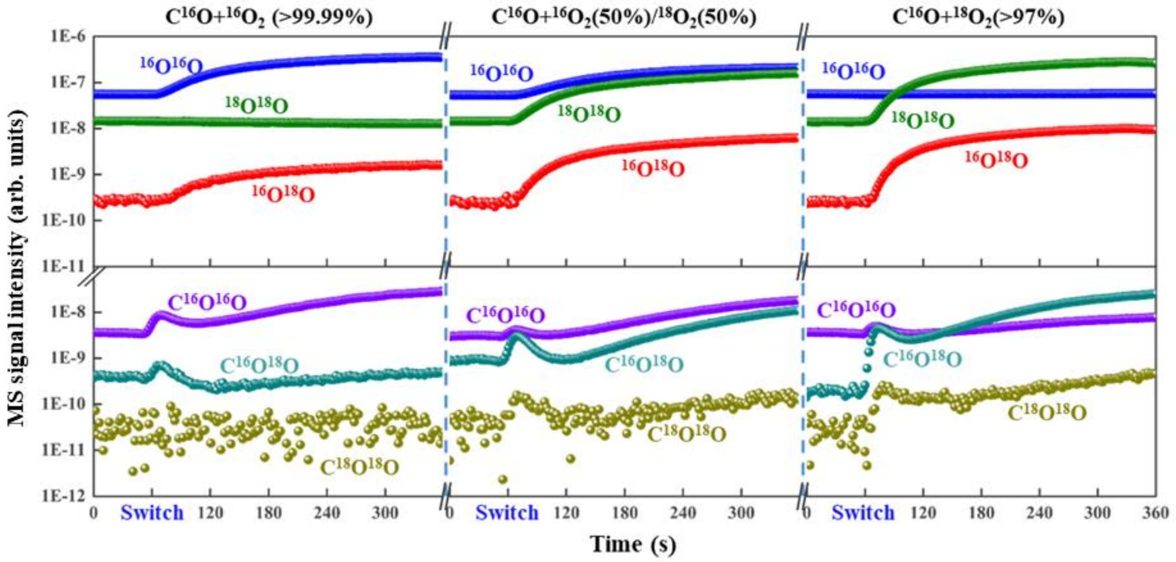


**Supplementary Figure 19.** Mass spectrometry (MS) data collected for the Pt/CNT-0 catalyst during the switch from Ar to Ar+$C^{16}O$+$^{16}O_2$ (>99.9%), Ar+$C^{16}O$+$^{16}O_2$ (50%)/$^{18}O_2$ (50%), and Ar+$C^{16}O$+$^{18}O_2$ (>97%). Reaction conditions: 140 ºC, ambient pressure, $P_{CO}$:$P_{O2}$:$P_{Ar}$=1:20:79, 60000 mL·$g_{cat}^{-1}$·$h^{-1}$.

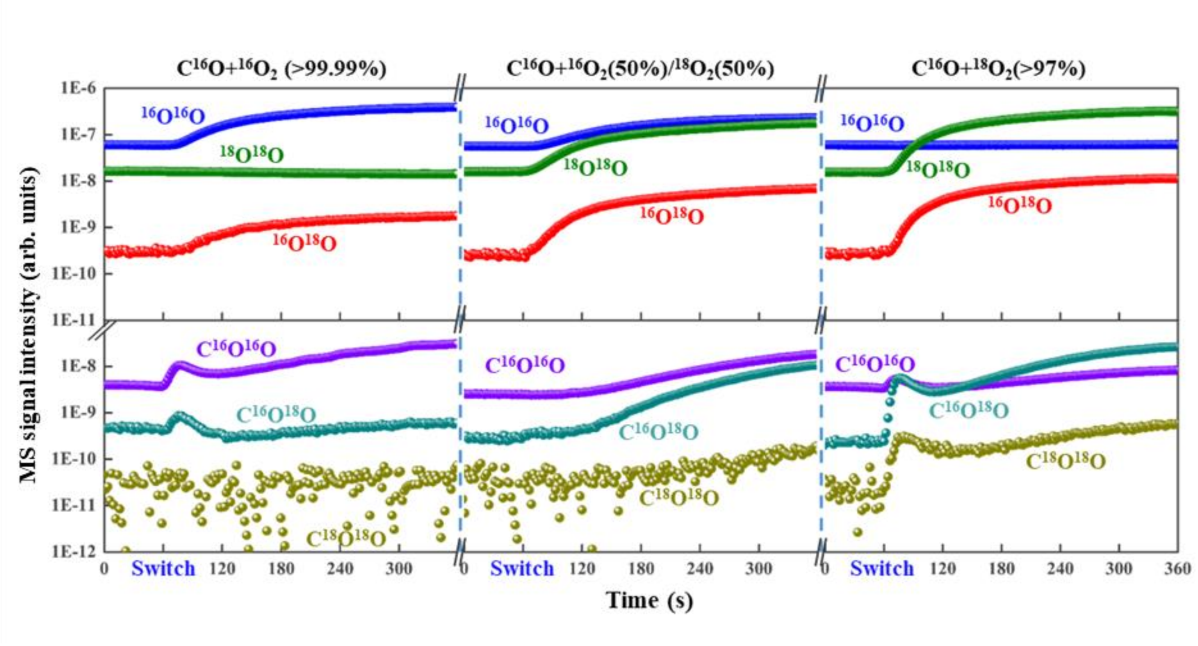


**Supplementary Figure 20.** Mass spectrometry (MS) data collected for the Pt/CNT-200 catalyst during the switch from Ar to Ar+$C^{16}O$+$^{16}O_2$ (>99.9%), Ar+$C^{16}O$+$^{16}O_2$ (50%)/$^{18}O_2$ (50%), and Ar+$C^{16}O$+$^{18}O_2$ (>97%). Reaction conditions: 140 °C, ambient pressure, $P_{CO}$:$P_{O2}$:$P_{Ar}$=1:20:79, 60000 mL·$g_{cat}^{-1}$·$h^{-1}$.

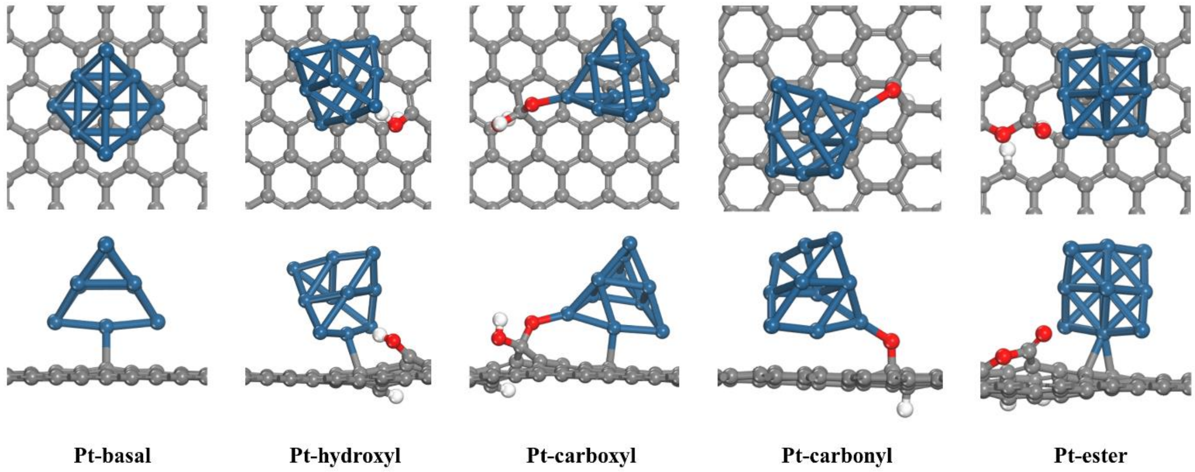


**Supplementary Figure 21.** The optimized structures for Pt-basal, Pt-hydroxyl, Pt-carboxyl, Pt-carbonyl, and Pt-ester.

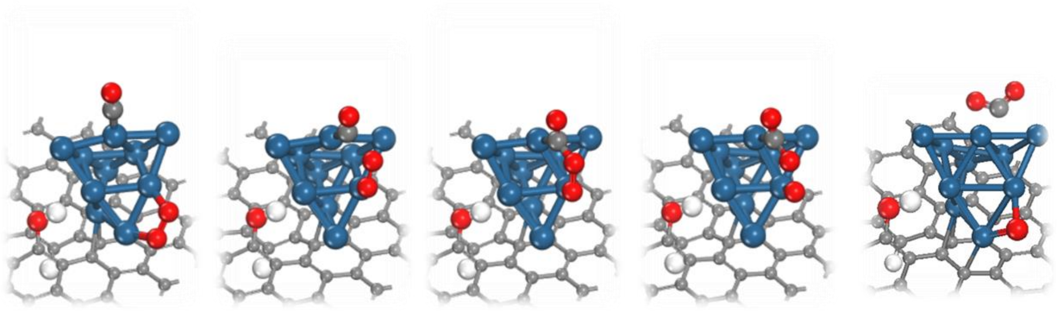

IS TS1 MS TS2 FS

**Supplementary Figure 22.** Optimized configurations of all reaction intermediates involved in the pathway of CO oxidation on Pt-hydroxyl. (gray: carbon, red: oxygen; blue: platinum; white: hydrogen).

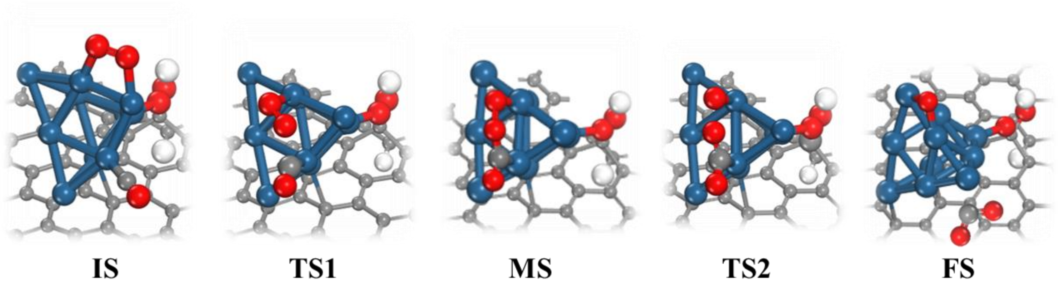


**Supplementary Figure 23.** Optimized configurations of all reaction intermediates involved in the pathway of CO oxidation on Pt-carboxyl. (gray: carbon, red: oxygen; blue: platinum; white: hydrogen).

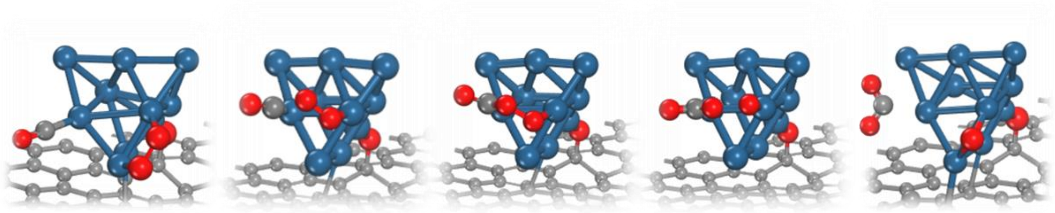


**Supplementary Figure 24.** Optimized configurations of all reaction intermediates involved in the pathway of CO oxidation on Pt-carbonyl. (gray: carbon, red: oxygen; blue: platinum; white: hydrogen).

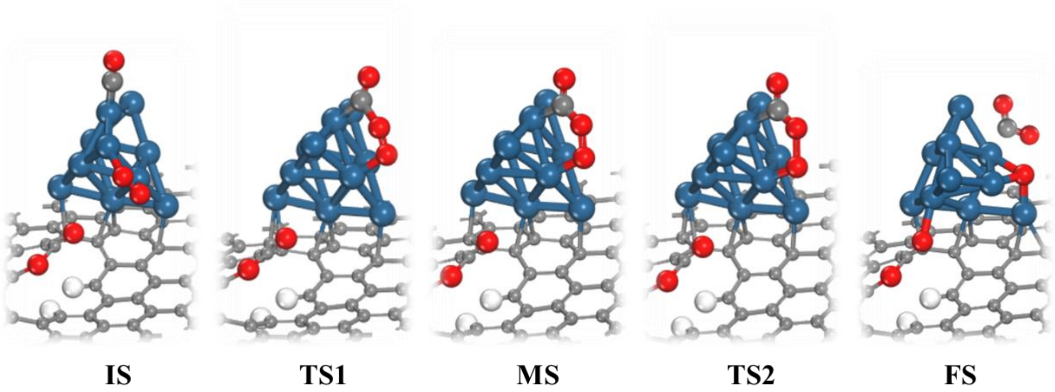


**Supplementary Figure 25.** Optimized configurations of all reaction intermediates involved in the pathway of CO oxidation on Pt-ester. (gray: carbon, red: oxygen; blue: platinum; white: hydrogen).

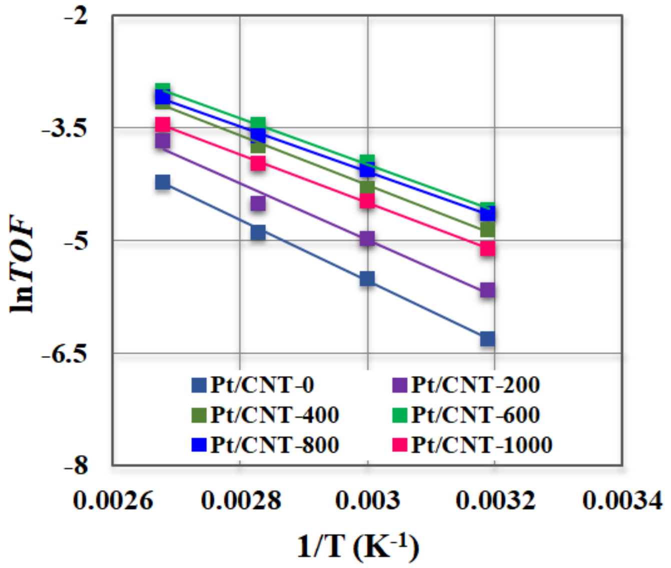


**Supplementary Figure 26.** The Arrhenius plots between ln TOF and 1/T for Pt/CNT-0, Pt/CNT-200, Pt/CNT-400, Pt/CNT-600, Pt/CNT-800 and Pt/CNT-1000.

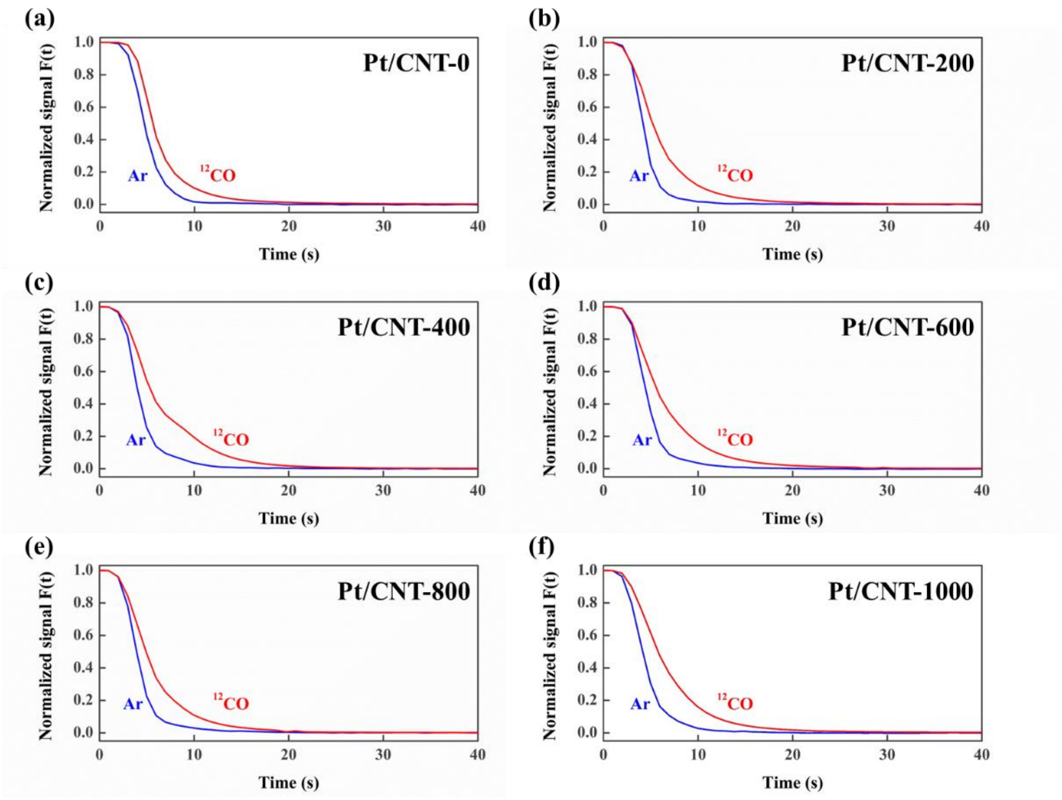


**Supplementary Figure 27.** The steady-state isotopic transients from Ar/$^{12}CO/O_2$ to Kr/$^{13}CO/O_2$ for (a) Pt/CNT-0, (b) Pt/CNT-200, (c) Pt/CNT-400, (d) Pt/CNT-600, (e) Pt/CNT-800 and (f) Pt/CNT-1000. (100 °C, $P_{CO}$:$P_{O2}$:$P_{Ar}$=1:20:79, 60000 mL·$g_{cat}^{-1}$·$h^{-1}$, and 1.85 bar).

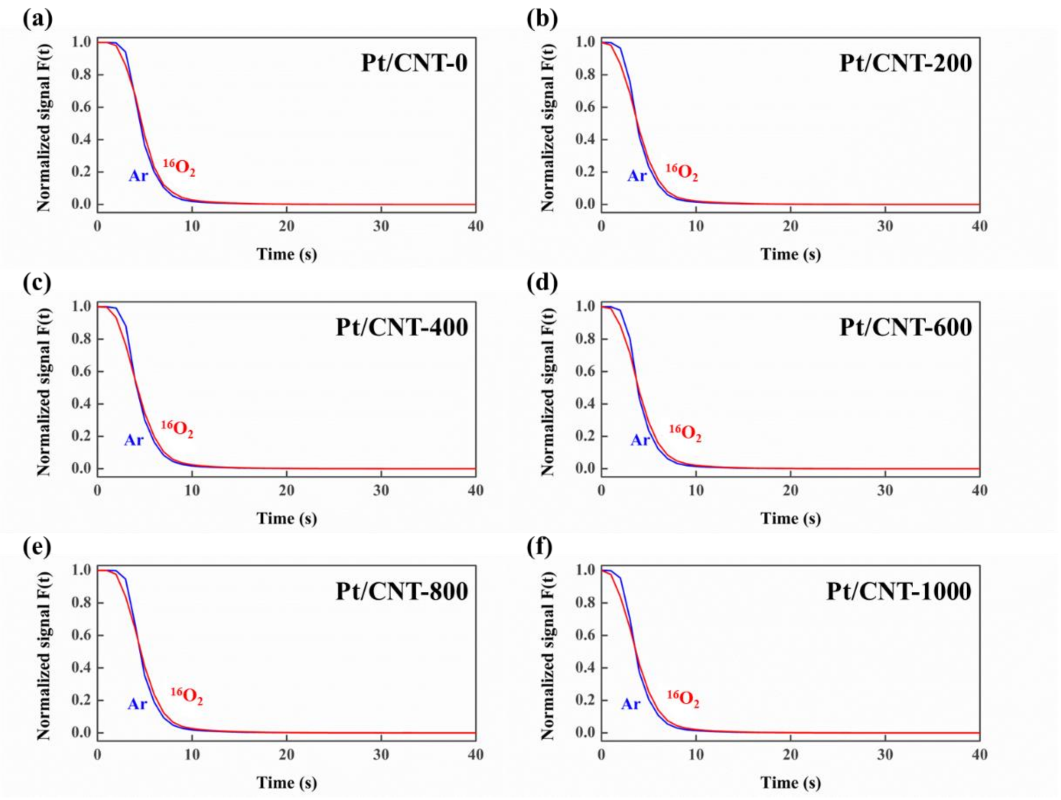


**Supplementary Figure 28.** The steady-state isotopic transients of Ar and $^{16}O_2$ following a switch from Ar/CO/$^{16}O_2$ to Kr/CO/$^{18}O_2$ for (a) Pt/CNT-0, (b) Pt/CNT-200, (c) Pt/CNT-400, (d) Pt/CNT-600, (e) Pt/CNT-800 and (f) Pt/CNT-1000. (100 °C, $P_{CO}$:$P_{O2}$:$P_{Ar}$=1:20:79, 60000 mL·$g_{cat}^{-1}$·$h^{-1}$, and 1.85 bar).

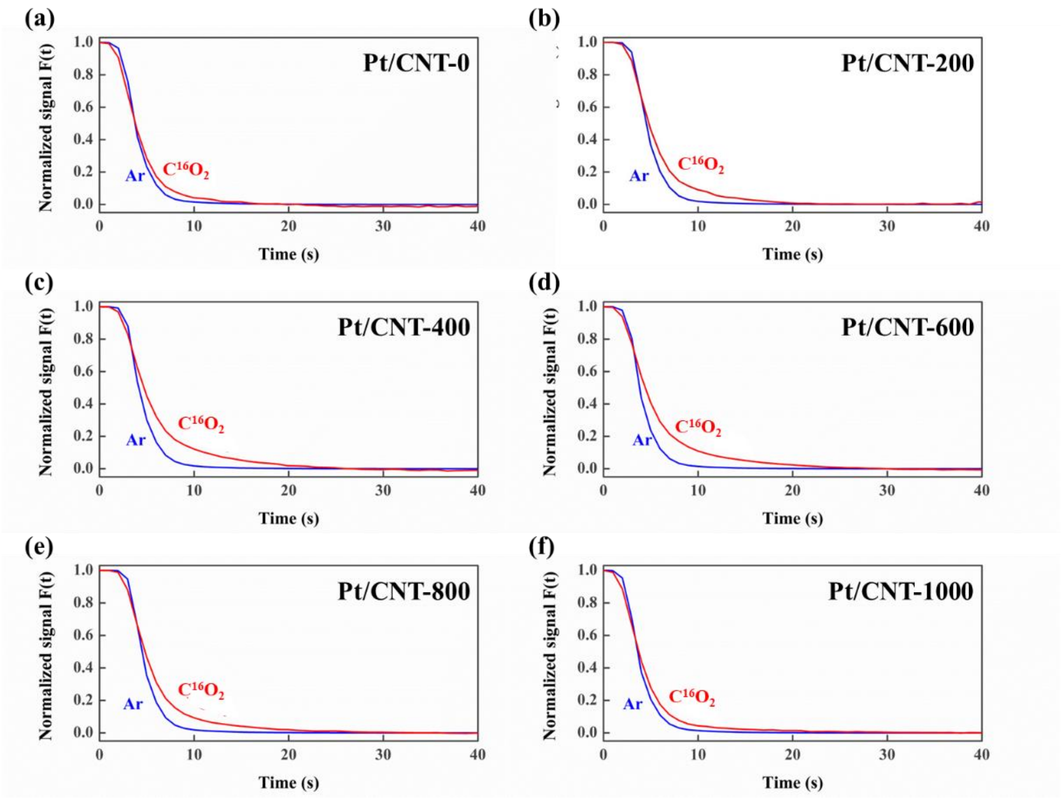


**Supplementary Figure 29.** The steady-state isotopic transients of Ar and $C^{16}O_2$ following a switch from $Ar/CO/^{16}O_2$ to $Kr/CO/^{18}O_2$ for (a) Pt/CNT-0, (b) Pt/CNT-200, (c) Pt/CNT-400, (d) Pt/CNT-600, (e) Pt/CNT-800 and (f) Pt/CNT-1000. (100 °C, $P_{CO}$:$P_{O2}$:$P_{Ar}$=1:20:79, 60000 $mL \cdot g_{cat}^{-1} \cdot h^{-1}$, and 1.85 bar).

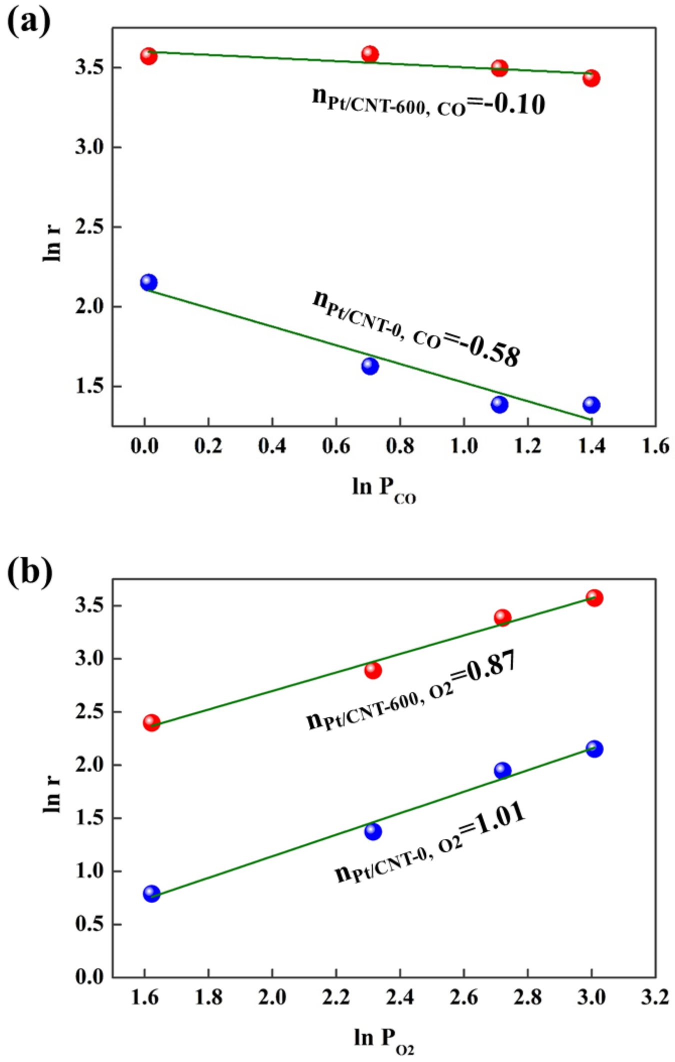


**Supplementary Figure 30.** The kinetic reaction orders of (a) CO and (b) $O_2$ for Pt/CNT-0 and Pt/CNT-600. The unit of pressure is KPa.

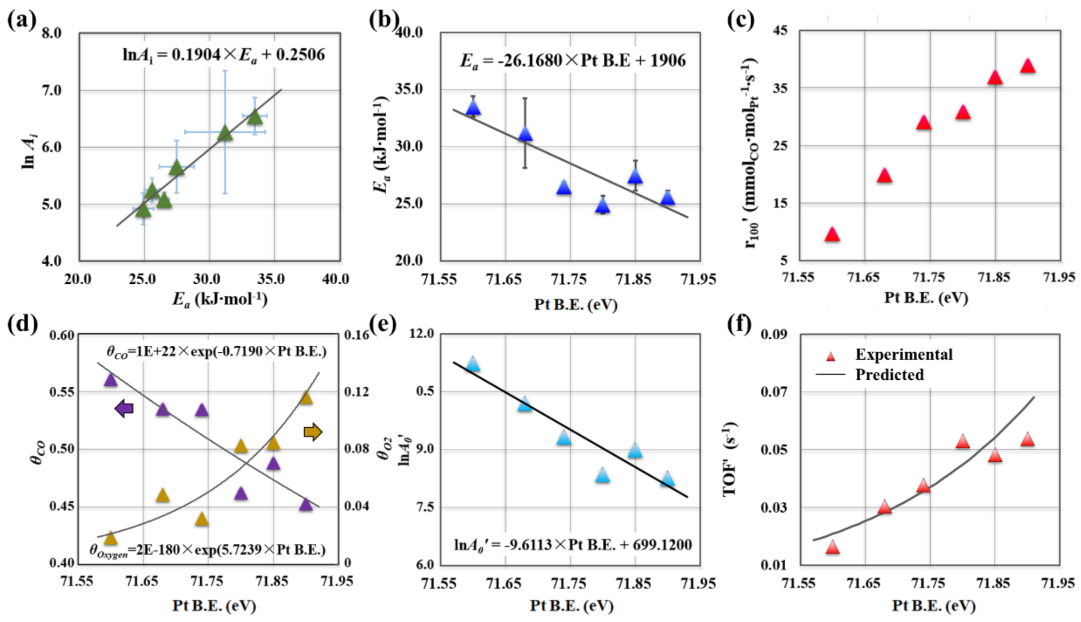


**Supplementary Figure 31.** The relationship between (a) ln$A_i$ and $E_a$, as well as (b) $E_a$, (c) $r_{100}$', (d) $\theta_{CO}$ and $\theta_O$, (e) ln$k_0'$, (f) the experimental and predicted TOF′ as a function of Pt B.E. for Pt/CNT-0, Pt/CNT-200, Pt/CNT-400, Pt/CNT-600, Pt/CNT-800 and Pt/CNT-1000. Reaction conditions: 100 °C, $P_{CO}$:$P_{O2}$:$P_{Ar}$=1:20:79, and 60000 mL·$g_{cat}^{-1}$·$h^{-1}$. Error bars in (a) and (b) were calculated from the standard error of each linear fit presented in Supplementary Fig. 26.
